# Metal‐Organic Framework Nanosheets as Templates to Enhance Performance in Semi‐Crystalline Organic Photovoltaic Cells

**DOI:** 10.1002/advs.202200366

**Published:** 2022-05-22

**Authors:** Kezia Sasitharan, Rachel C. Kilbride, Emma L.K. Spooner, Jenny Clark, Ahmed Iraqi, David G. Lidzey, Jonathan A. Foster

**Affiliations:** ^1^ Department of Chemistry The University of Sheffield Dainton Building, Brook Hill Sheffield S3 7HF UK; ^2^ Department of Physics and Astronomy The University of Sheffield Hicks Building, Hounsfield Road Sheffield S3 7RH UK

**Keywords:** Two dimensional materials, bulk heterojunctions, metal‐organic framework nanosheets, organic photovoltaic devices, organic solar cells

## Abstract

Optimizing the orientation, crystallinity, and domain size of components within organic photovoltaic (OPV) devices is key to maximizing their performance. Here a broadly applicable approach for enhancing the morphology of bulk heterojunction OPV devices using metal–organic nanosheets (MONs) as additives is demonstrated. It is shown that addition of porphyrin‐based MONs to devices with fully amorphous donor polymers lead to small improvements in performance attributed to increased light absorption due to nanosheets. However, devices based on semi‐crystalline polymers show remarkable improvements in power conversion efficiency (PCE), more than doubling in some cases compared to reference devices without nanosheets. In particular, this approach led to the development of PffBT4T2OD‐MON‐PCBM device with a PCE of 12.3%, which to the authors’ knowledge is the highest performing fullerene based OPV device reported in literature to date. Detailed analysis of these devices shows that the presence of the nanosheets results in a higher fraction of face‐on oriented polymer crystals in the films. These results therefore demonstrate the potential of this highly tunable class of two‐dimensional nanomaterials as additives for enhancing the morphology, and therefore performance, of semi‐crystalline organic electronic devices.

## Graphical abstract

Porphyrin based metal‐organic framework nanosheets are added to a range of organic photovoltaic devices resulting in the highest performing fullerene based devices reported to date. Analysis shows that for devices based on semi‐crystalline polymers, the nanosheets increase the fraction of face‐on oriented polymer crystals in the films leading to enhanced absorbance and charge transport.

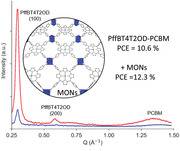

## 1 Introduction

Organic photovoltaics (OPV) offer many potential advantages over current silicon technologies such as low‐cost production, large area manufacturing capability, and the potential to create flexible and semi‐transparent devices.^[^ ^1^, ^2^ ^]^ This field has progressed rapidly over the last 5 years with improved donor polymers,^[^ ^3^, ^4^ ^]^ novel non‐fullerene acceptors,^[^ ^5^, ^6^ ^]^ and improved hole transport layers, leading to record power conversion efficiencies (PCE) > 18%.^[^ ^4^, ^5^, ^6^, ^7^, ^8^, ^9^, ^10^, ^11^ ^]^ However, the absorption, charge transport, and stability of the components within the devices depend not only on their molecular structure but also on the way they pack together. New approaches to controlling orientation, crystallinity, and domain size within organic electronic devices are therefore key to maximizing the performance of this promising class of devices.^[^ ^9^, ^12^, ^13^ ^]^

The photoactive layers in a bulk heterojunction (BHJ) OPV device typically consists of an interpenetrating network of electron donating polymers and electron accepting molecules.^[^ ^14^, ^15^, ^16^ ^]^ Both components become finely mixed at the nanoscale level during the film‐casting process. Previous studies have shown that an optimal morphology is the one where the donor polymer is oriented in the direction that provides a higher charge mobility, typically in the direction of *π*–*π* stacking.^[^ ^17^, ^18^, ^19^, ^20^ ^]^ Higher charge mobility can also be achieved by increasing the crystallinity of the donor polymer.^[^ ^21^ ^]^ Crystallinity is also known to be a key determinant of stability for a broad range of polymers. This is because the amorphous regions are known to contain more triplet states that lead to faster device degradation.^[^ ^22^ ^]^ Typical approaches to control the nanoscale morphology and improve the film crystallinity within the OPV devices include thermal annealing, solvent annealing,^[^ ^23^ ^]^ and the use of solvent additives.^[^ ^24^ ^]^ Graphene and other low dimensional nanomaterials have found a wide range of use in solar cells, including as ternary components within the active layer.^[^ ^25^ ^]^ However, the simple inorganic structures of most of these materials mean their optical and electronic properties are inherently linked to their surface chemistry making them difficult to optimize independently.

Metal‐organic nanosheets (MONs) are graphene‐like 2D materials, composed of organic linkers coordinated to metal‐ions or clusters.^[^ ^26^, ^27^, ^28^ ^]^ These materials combine the high surface areas and nanoscale dimensions of 2D materials with a modular structure that allows for easy tuning of their optoelectronic properties, porosity, and surface chemistry.^[^ ^29^ ^]^ These properties have enabled promising progress in the use of MONs in separation, catalysis, sensing, and most recently in electronic devices including sensors, batteries, supercapacitors, and light emitting diodes.^[^ ^27^, ^28^, ^30^, ^31^, ^32^, ^33^ ^]^ To our knowledge, there are currently four papers which show how MONs can be used within solar cell applications in different capacities. Huang and co‐workers mixed tellurophene based MONs with ZnO and showed they could be used as electron extraction layer into organic solar cells.^[^ ^34^ ^]^ Liu et al. used a Langmuir–Blodgett approach to deposit a film of porphyrin based MONs onto ITO and infilled them with C_60_ to form a liquid junction solar cell.^[^ ^35^ ^]^ Most recently, A.K.Y Jen and co‐workers demonstrated the use of MONs as an electron extraction layer at the perovskite/cathode interface reducing the leakage of toxic lead ions and so improving device stability.^[^ ^36^ ^]^

We recently reported the first example showing incorporation of MONs into the photoactive layer of OPV BHJs.^[^ ^37^ ^]^ Porphyrin MONs were added to the archetypal P3HT‐PCBM architecture through a simple spin‐coating method to create a ternary blend solar cell. Remarkably, the addition of MONs resulted in a near doubling in device PCE from 2.7% to 5.2%.^[^ ^37^ ^]^ Detailed investigations into the morphology of the active layer showed that the relative proportion of crystalline regions in the thin films is improved upon incorporation of MONs. This led to enhanced absorption and more balanced charge mobility resulting in simultaneous improvements in both the fill‐factor (FF) and current density (*J*_SC_).

Based on these results, we hypothesized that similar improvements in performance might be observed in other OPV devices based on semi‐crystalline polymers. Here we therefore investigate the effect of adding MONs to the active layer of six different OPV devices to better understand the role of nanosheets and create higher performing devices. We initially evaluated devices based on amorphous and semi‐crystalline donor polymers and with different fullerene acceptors in order to understand the impact of the nanosheets in different systems. In the process we created the highest performing fullerene‐based OPV device reported in the literature. We then undertook detailed analysis of the properties and morphology of these devices in order to understand the role of the nanosheets. Finally, we discuss how the insights obtained through this study might be applicable to improve other organic electronic devices.

## 2 Results

**Figure 1:**
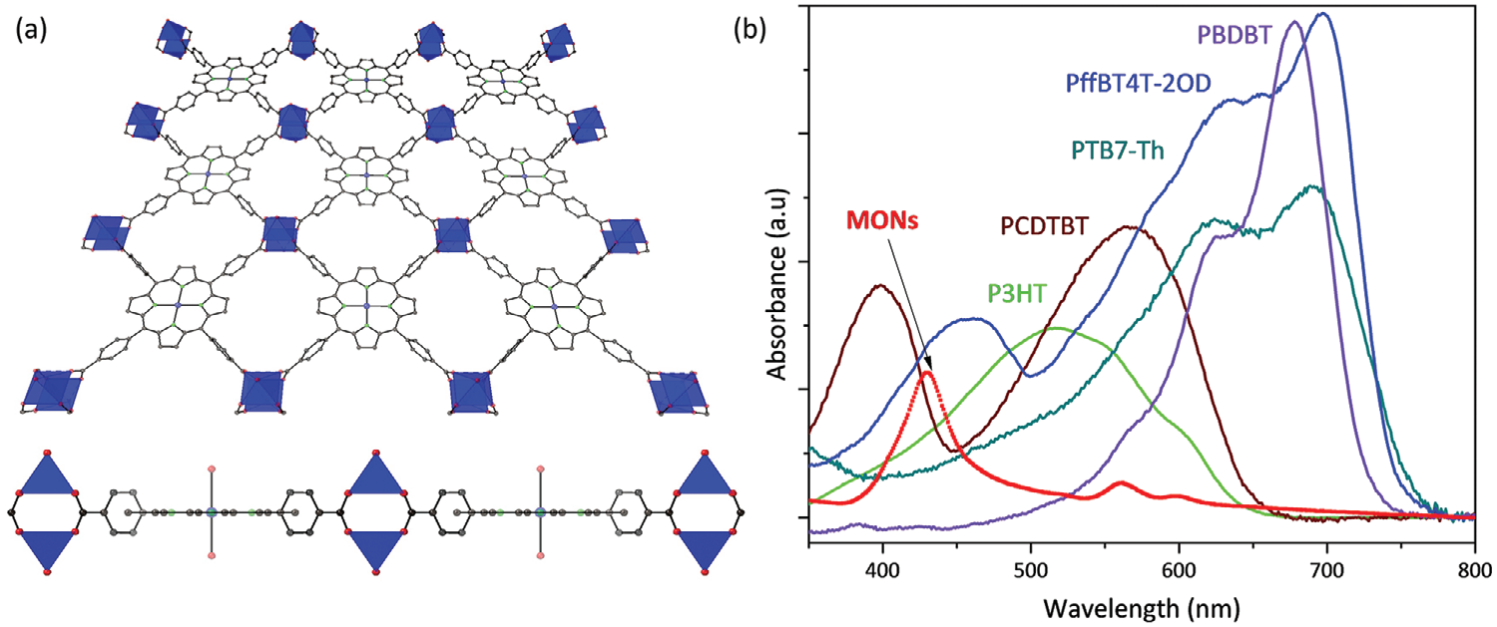
a) Crystal structure showing a single layer of ZnTCPP from different angles—each TCPP linker is connected to four Zn paddlewheel units to form an extended 2D structure. b) Thin film absorption spectra of the MONs and the various polymers used in this study (spin‐coated on ITO coated glass).

Nanosheets of Zn_2_(ZnTCPP), where TCPP is tetrakis(4‐carboxyphenyl) porphyrin, were synthesized according to a previously reported procedure.^[^ ^38^ ^]^ For this work, twenty parallel syntheses of 5 mg metal–organic framework (MOF) in 5 mL of ethanol were exfoliated in an ultrasonic bath for 60 min and then centrifuged for 10 min at 1500 rpm. The samples were then combined, the solvent evaporated and stored as a powder until needed. As shown in **Figure** **1**a, the MONs consist of TCPP ligands connected to four zinc paddlewheel secondary building units to create an extended structure.^[^ ^37^ ^]^ UV–vis spectroscopy shows that the absorption of the porphyrin nanosheets overlaps well with the visible region of the solar spectrum and are complementary to some of the most commonly employed OPV polymers making them promising ternary components (Figure 1b).

The devices in this work were prepared with an inverted architecture and the different layer stacks are ITO:ZnO:Active layer:MoO_3_:Ag. To test the photovoltaic properties, *J–V* curves were recorded by illuminating the devices using a Newport AM 1.5 solar simulator calibrated against an NREL standard silicon solar cell. In all cases, an aperture mask was placed on top of the device to ensure the light exposed area of the device was limited to 2.6 mm^2^.

Ternary blends with between 5 and 20 wt% of MON were produced by re‐dispersing a weighed amount of MONs into chlorobenzene through sonication for 10 min. This suspension was then added to the various polymer‐fullerene combinations prior to spin‐coating. The supporting information lists the device preparation and the optimization of the percentage by weight loading of MONs in each type of active layer. Key metrics for the “champion” devices and the standard deviation on 20 devices with and without MONs are detailed in **Table** **1**.

**Table 1:** OPV device performance metrics for the champion (best‐performing) device and the average values with their standard deviation

Active layer	*J*_SC_ [mA cm^−2^]	*V*_OC_ [V]	FF [%]	PCE [%]	ΔPCE [%]
P3HT‐PCBM^a)^ Semi‐crystalline	7.09 7.09 ± 0.15	0.66 0.66 ± 0.02	57.0 57.4 ± 2.2	2.7 2.6 ± 0.01	
P3HT‐MON‐PCBM^a)^ Semi‐crystalline	10.8 10.54 ± 0.2	0.69 0.70 ± 0.03	69.0 63.1 ± 0.2	5.2 4.6 ± 0.3	+2.5
P3HT‐ICBA Semi‐crystalline	6.23 6.0 ± 0.23	0.87 0.87 ± 0.01	56.1 55.5 ± 0.5	3.0 2.8 ± 0.3	
P3HT‐MON‐ICBA Semi‐crystalline	12.0 12.0 ± 0.02	0.85 0.85 ± 0.01	60.0 59 ± 0.1	6.1 5.9 ± 0.1	+3.1
PCDTBT‐PCBM Amorphous	9.07 8.5 ± 0.5	0.89 0.89 ± 0.01	67.3 67.2 ± 0.1	5.4 5.2 ± 0.2	
PCDTBT‐MON‐PCBM Amorphous	9.54 9.2 ± 0.3	0.89 0.89 ± 0.01	66.3 66.3 ± 0.02	5.6 5.4 ± 0.2	+0.2
PTB7Th‐PCBM Amorphous	13.08 12.9 ± 0.1	0.83 0.83 ± 0.02	56.0 56 ± 0.01	6.1 6.0 ± 0.1	
PTB7Th‐MON‐PCBM Amorphous	13.32 13.14 ± 0.2	0.84 0.84 ± 0.02	58.9 58.4 ± 0.5	6.6 6.2 ± 0.4	+0.5
PBDBT‐PCBM Amorphous	12.5 12.2 ± 0.3	0.89 0.89 ± 0.01	63.7 63.6 ± 0.07	7.1 7.0 ± 0.08	
PBDBT‐MON‐PCBM Amorphous	13.7 13.6 ± 0.1	0.89 0.89 ± 0.02	64.1 63.6 ± 0.07	7.8 7.4 ± 0.4	+0.7
PffBT4T2OD‐PCBM Semi‐crystalline	19.0 18 ± 1.02	0.77 0.77 ± 0.01	72.4 71.5 ± 0.9	10.6 9.7 ± 0.9	
PffBT4T2OD‐MON‐PCBM Semi‐crystalline	21.6 21.2 ± 0.41	0.77 0.77 ± 0.01	74.0 73.7 ± 0.3	12.3 11.9 ± 0.3	+1.7

Acronyms used in this study: PCBM = phenyl‐C61‐butyric acid methyl ester, P3HT = poly(3‐hexylthiophene), ICBA = 1′,1′′,4′,4′′‐Tetrahydro‐di[1,4]methanonaphthaleno[1,2:2′,3′,56,60:2′′,3′′][5,6]fullerene‐C60, PCDTBT = poly[N‐9″‐hepta‐decanyl‐2,7‐carbazole‐alt‐5,5‐(4′,7′‐di‐2‐thienyl‐2′,1′,3′‐benzothiadiazole)], PBT7Th = poly[4,8‐bis(5‐(2‐ethylhexyl)thiophen‐2‐yl)benzo[1,2‐b;4,5‐b′]dithiophene‐2,6‐diyl‐alt‐(4‐(2‐ethylhexyl)‐3‐fluorothieno[3,4‐b]thiophene‐)‐2‐carboxylate‐2‐6‐diyl], PBDB‐T = poly[(2,6‐(4,8‐bis(5‐(2‐ethylhexyl)thiophen‐2‐yl)benzo[1,2‐b:4,5‐b′]dithiophene)‐co‐(1,3‐di(5‐thiophene‐2‐yl)‐5,7‐bis(2‐ethylhexyl)benzo[1,2‐c:4,5‐c′]dithiophene‐4,8‐dione)], PCE11 = PffBT4T‐2OD = poly[(5,6‐ difluoro‐2,1,3‐benzothiadiazol‐4,7‐diyl)‐alt‐(3,3′′′‐di(2‐octyldodecyl)‐2,2′;5′,2″;5″,2′′′‐quaterthiophen‐5,5′′′‐diyl)];

^a)^ Systems Reproduced under the terms of the Creative Commons CC BY license.^[^ ^35^ ^]^ Copyright 2019, The Authors, published by Royal Society of Chemistry (NOTE: These solar cells were of conventional architecture with PEDOT:PSS and BCP/Ag as the hole transport and electron transport layers, respectively).

P3HT is the prototypical semicrystalline polymer which we used in our previous studies alongside the archetypal fullerene acceptor, PCBM.^[^ ^37^ ^]^ We found that addition of 20% MONs resulted in a PCE of 5.2%, almost twice that for reference devices without nanosheets with a simultaneous improvement of *J*_SC_, *V*_OC_, and FF. Detailed analysis showed addition of the nanosheets resulted in an increase in the crystalline fraction of P3HT which lead to a doubling of the absorbance and a tenfold increase in hole mobility.

**Figure 2:**
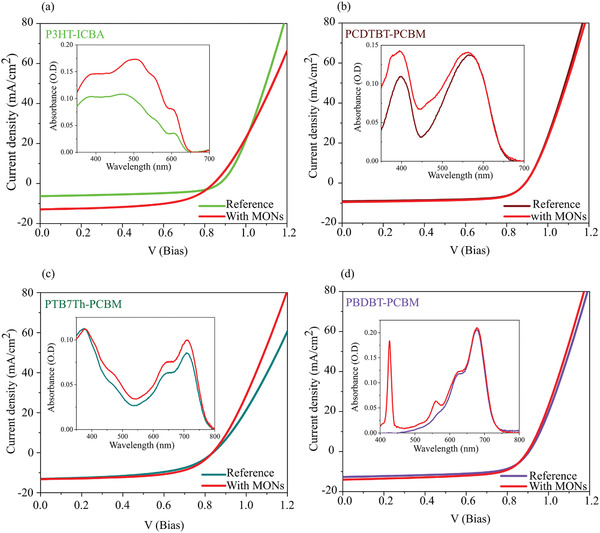
*J–V* characteristics of the photovoltaic devices and inset—absorption spectra of a) P3HT:ICBA, b) PCDTBT:PCBM, c) PTB7Th:PCBM, and d) PBDBT:PCBM, with and without MONs.

We sought to build on these initial studies by incorporating an improved fullerene acceptor to create P3HT‐ICBA devices. Incorporation of 20 wt% of MONs into the active layer of these devices resulted in the PCE more than doubling from 3.04% to 6.10% (**Figure** **2**a). The improvement in PCE was accompanied by an increase in the *J*_SC_ of the devices by 6 mA cm^−2^ and fill factor by about 4% compared to those without MONs. The *V*_OC_ remained unaffected with the addition of MONs. Analysis of the absorption spectra of the films showed an enhancement of the absorption coefficient upon incorporation of MONs. The increase in absorption coefficient in the wavelength range 430–450 nm corresponds to the *π*–π* transition absorption of MONs and the enhancement in the 550–580 nm range corresponds to the Q absorption bands of the MONs. Increased absorbance in the 600–630 nm corresponds to an increased percentage of crystalline P3HT structures. These observations match with our previous detailed investigations on the P3HT‐PCBM films, where MONs improved the polymer crystallinity.^[^ ^37^ ^]^

PCDTBT is a representative amorphous BHJ system with a bilayer lamellar arrangement.^[^ ^39^ ^]^ In contrast to the P3HT‐PCBM and P3HT‐ICBA results, no pronounced enhancement in PCE was observed upon incorporation of MONs into amorphous PCDTBT‐PCBM devices (Figure 2b). At 10 wt% loading of MONs, the *J*_SC_ increases marginally by 0.5 mA cm^−2^. The *V*_OC_ remained unchanged, the FF decreased by 1% and the overall PCE of the highest performing device remained 5.5%. PCDTBT has been reported to show a limited short range order due to *π*–*π* stacking between polymers normal to the plane of the substrate.^[^ ^39^ ^]^ The nanosheets are expected to be orientated parallel to the substrate plane which could explain why no templating effect is observed in this case. The optical density was found to increase slightly in the wavelength range 400–550 nm, which matched the absorption profile of the porphyrin units in the MON structure. We attribute the increase in *J*_SC_ that offset the decreased FF to this increased light absorbance by the MONs.

PTB7Th polymer is a donor–acceptor type system reported as consisting of amorphous phases intermixed in largely disordered aggregates.^[^ ^40^ ^]^ PTB7Th‐PCBM devices showed small, but not statistically significant improvements to the device PCE upon incorporation of MONs. At 5% weight loading of MONs in PTB7Th‐PCBM devices, the *J*_SC_ and FF improved by 0.3 mA cm^−2^ and ≈3% respectively, whilst the *V*_OC_ remained unchanged leading to an increase in PCE from 6% to 6.5% (Figure 2c). A few previous literature reports on PTB7‐Th have shown this class of polymers can occasionally result in improved aggregation features when combined with highly crystalline small molecules and other additives.^[^ ^21^, ^40^, ^41^, ^42^, ^43^, ^44^, ^45^, ^46^, ^47^, ^48^, ^49^, ^50^ ^]^ However, morphological and chain packing studies on these polymers support the existence of a major fraction of amorphous regions in the photoactive layer with very short correlation lengths, similar to PCDTBT.^[^ ^40^, ^43^, ^51^, ^52^ ^]^ The absorption spectra for the devices with MONs showed a slight increase in the wavelength region 400–750 nm. Since the porphyrins only absorb up to 550 nm, it is unclear whether the increased absorbance in the longer wavelength region arises from thickness effects in the film or from changes to film crystallinity.

PBDBT is a rigid semi‐conducting polymer with complex morphology which has been variously characterized as crystalline,^[^ ^53^, ^54^ ^]^ amorphous,^[^ ^55^ ^]^ and most recently as semi‐paracrystalline.^[^ ^56^ ^]^ PBDBT‐PCBM devices showed an increase in PCE from 7.1% to 7.8% upon incorporation of a 5 wt% loading of MONs. This was accompanied by an underlying increase in *J*_SC_ by 1 mA cm^−2^ and FF by 1% with no change in *V*_OC_. This polymer does not absorb in the porphyrin absorption region, hence the contribution from the S‐band of MON in the absorption spectra at 430 nm was clearly observed (Figure 2d). The Q‐absorption band of porphyrins at 564 nm was also observed, contributing to a further increase in absorbance intensity. These findings explained the 1 mA cm^−2^ increase in *J*_SC_, the second highest increase (after P3HT) among the polymers investigated so far.

**Figure 3:**
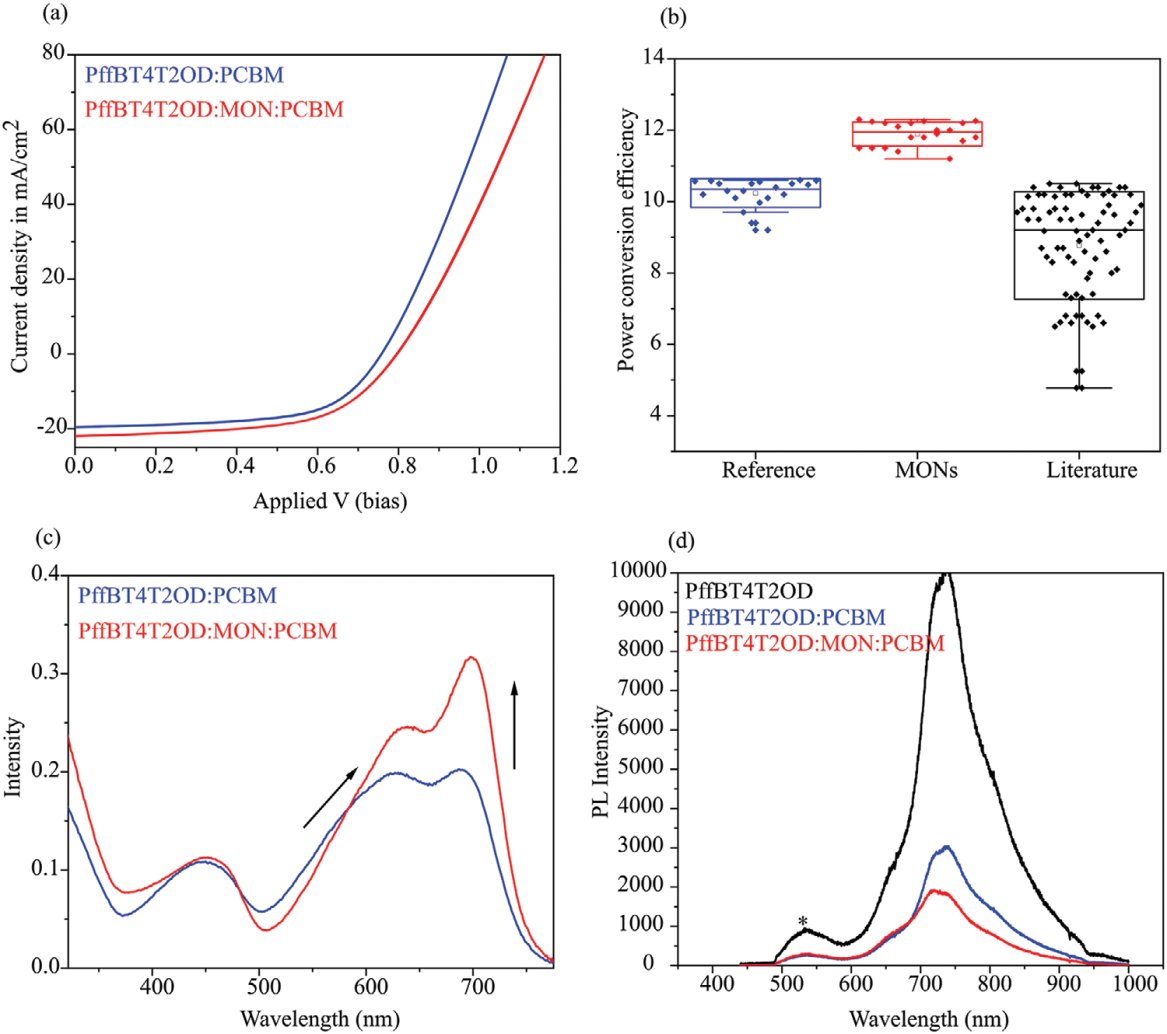
a) *J–V* curve of PffBT4T2OD:PCBM and PffBT4T2OD:MON:PCBM devices. b) Box plots showing the distribution of the PCEs obtained from the 20 reference and 20 MON based devices that were prepared in this study to check the statistical significance of the results and enhancement in performance. c) Absorbance spectra of thin‐films of PffBT4T2OD:PCBM and PffBT4T2OD:MON:PCBM. d) Photoluminescence spectra of the PffBT4T2OD:PCBM and PffBT4T2OD:MON:PCBM films. *Residual excitation.

PffBT4T2OD (often referred to as PCE11) is a semi‐crystalline polymer which exhibits temperature dependent aggregation behavior with the ability to be engineered into highly crystalline domains.^[^ ^22^ ^]^ PffBT4T2OD:PCBM devices showed a remarkable increase in performance parameters upon incorporation of MONs with a PCE of 12.3% for the champion device as compared to 10.6% for the reference device (**Figure** **3**a). The *J*_SC_ increased by 3 mA cm^−2^, the fill factor increased by 2%, and the *V*_OC_ remained unaffected within error. Figure 3b shows the statistical analysis of the PCE (%) obtained from 20 reference devices and 20 MON based devices prepared in this work. We have also analyzed the works in literature that have reported OPV devices based on this PffBT4T2OD polymer to test the significance of this enhancement that MONs have brought about. Table S1, Supporting Information lists the literature values, references, and the methodology used for the literature search. Upon comparing with the results from 47 literature reports, we found that our MON incorporated PffBT4T2OD:PCBM devices with a mean PCE of 11.9 ± 0.3% are the highest reported so far for this class of polymer. Our champion device at 12.3% is also (to the best of our knowledge) the best fullerene based OPV so far reported.

The PffBT4T2OD system was therefore chosen for further investigations into the mechanism of performance enhancement. A comparison of the thin‐film absorption spectra (Figure 3c) of the pristine polymer with that of a polymer‐MON blend was undertaken to better understand the effect of MONs on light absorption. The polymer has a characteristic absorption spectrum with bands at 450, 560, and 700 nm. In the presence of the MONs, the vibronic peak at 560 and 700 nm are red shifted, indicating an increase in conjugation length, which is accompanied by an increase in the intensity of the peak at 700 nm. These features can be attributed to the formation of aggregates (short‐ranged ordered moieties) in the film.^[^ ^57^, ^58^ ^]^

Charge transfer between PffBT4T2OD and PCBM has previously been reported.^[^ ^59^, ^60^ ^]^ Photoluminescence (PL) quenching measurements were therefore undertaken in order to investigate the effect of MONs on charge separation at the donor–acceptor interface. PL spectra of neat PffBT4T2OD film and blends with PCBM, and MON:PCBM are shown in Figure 3d. By integrating the area under the curve, we found that the PL quenching with PCBM is only 75%, whereas when it was blended with MON:PCBM, the quenching efficiency was 90%. This enhanced quenching may be indicative of efficient charge transfer in the three‐component film, in which addition of MONs promotes charge separation and transport. This result was analogous to previous findings where optimized ternary blends exhibit better PL quenching than binary systems.^[^ ^60^, ^61^, ^62^ ^]^

The HOMO‐LUMO energy levels of the MONs have previously been derived and were compared to those reported for PffBT4T2OD and PCBM and used to construct an energy level diagram (Figure S12, Supporting Information). The band gap for the MONs was calculated to be larger than that of the donor polymer opening up the possibility of the MONs act as a sensitizer to extend the absorption range, with energy transfer to the donor polymer through a Dexter or FRET mechanism. A pre‐requisite for this energy transfer is that there should be significant overlap between the emission of the sensitizer (MON) and the absorption of the donor to allow efficient energy transfer. The MONs show a broad PL emission between 500 and 800 nm,^[^ ^37^ ^]^ and PffBT4T2OD absorbs between 400 and 750 nm with an absorption maxima at 700 nm (Figure 3c). Therefore, there is a significant overlap between the emission of MONs and absorption of the donor polymer to facilitate efficient energy transfer.

To better understand the influence of MONs on the charge carrier transport, space‐charge limited current (SCLC) measurements was utilized to estimate charge carrier mobilities. Section S9, Supporting information details measured *J–V* curves of hole‐only and electron‐only devices at room temperature. Hole‐only and electron‐only devices were prepared by adjusting the buffer layers, with dark injection curves fitted using the Mott–Gurney law.^[^ ^22^ ^]^ The average hole and electron mobility values based on three measurements for each sample are shown in **Table** **2**. After adding MONs, the hole mobility increased to 2.07 × 10^−3^ cm^2^ V^−1^s^−1^ which is 50% higher than the reference device (1.35 × 10^−3^ cm^2^ V^−1^s^−1^). The electron mobility was also found to increase by 60% after incorporation of MONs (from 1.40 × 10^−3^ to 2.29 × 10^−3^ cm^2^ V^−1^s^−1^). This improved mobility is partly responsible for the improved *J*_SC_ in the ternary blend OPVs and suggests that the addition of MONs has a favorable impact on the film microstructure and morphology.

**Table 2:** Average charge mobility values in cm^2^ V^−1^s^−1^ in binary and ternary OPVs, estimated using SCLC model based on three samples for each configuration

Device	Thickness	*μ*_h_ (Hole mobility)	*µ*_e_ (Electron mobility)	*μ*_h_/*µ*_e_
PffBT4T2OD/PCBM	150 nm	1.35 × 10^−3^	1.40 × 10^−3^	0.96
PffBT4T2OD/MON/PCBM	150 nm	2.07 × 10^−3^	2.29 × 10^−3^	0.90

**Figure 4:**
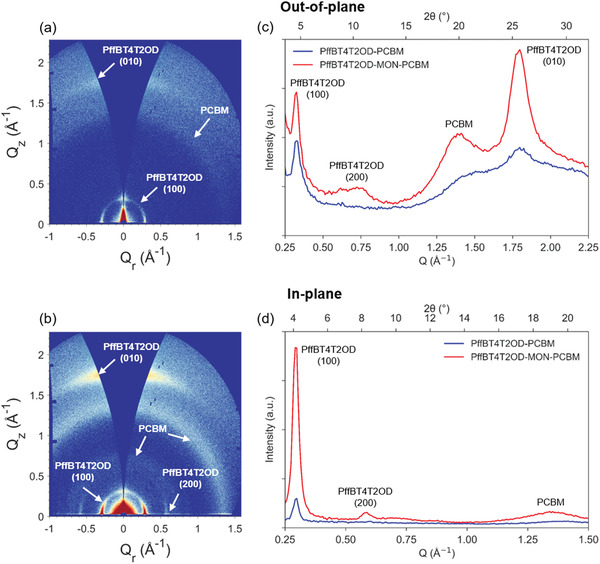
GIWAXS measurements on the active layer films a) PffBT4T2OD:PCBM and b) PffBT4T2OD:MON:PCBM films; comparison of azimuthally integrated 1D intensity profiles in the c) out of plane and d) in‐plane directions for films. A comparison of the in‐plane and out‐plane scattering intensities is provided in Figure S5, Supporting Information.

Grazing incidence wide angle X‐ray scattering (GIWAXS) studies were undertaken in order to investigate how the incorporation of MONs affects the molecular packing and orientation of the polymer chains within the thin‐films. Semi‐crystalline polymers typically pack into 2D sheets through face‐to‐face *π*–*π* interactions between the aromatic polymer backbones which then stack via their side chains into lamellar structures.^[^ ^63^, ^64^, ^65^ ^]^ The 2D GIWAXS patterns of the film with MONs show a huge enhancement in the out‐of‐plane *π*–*π* stacking intensity and the in‐plane lamellar intensity. In comparison, the 2D scattering features of the film without MONs are considerably weaker suggesting a lower degree of molecular order. To index and probe the orientation of the observed 2D scattering features, 1D azimuthal integrations were performed corresponding to the in‐plane and out‐of‐plane directions (**Figure** **4**a,b). A guide displaying the integration areas is provided in Figure S4, Supporting Information. Peaks were assigned on the basis of previous GIWAXS studies on PffBT4T‐2OD and are summarized in **Table** **3**.^[^ ^43^, ^66^, ^67^, ^68^, ^69^ ^]^

**Table 3:** GIWAXS peak indexing and Scherrer analysis for the out‐of‐plane and in‐plane azimuthally integrated 1D intensity profiles

	*Q* [Å^−1^]		*d*‐spacing $\left(d=\frac{2\pi }{Q}\right)$ [Å]		Crystal coherence length CCL^a)^ [a.u. nm]		Peak indexing
	PffBT4T2OD‐PCBM	PffBT4T2OD‐MON‐PCBM	PffBT4T2OD‐PCBM	PffBT4T2OD‐MON‐PCBM	PffBT4T2OD‐PCBM	PffBT4T2OD‐MON‐PCBM	
Out‐of‐plane	0.328	0.321	19.18	19.60	6.40	8.11	PffBT4T2OD lamellar (100)
	N/A	0.664	N/A	9.46	N/A	1.79	PCBM
	1.484	1.416	4.23	4.44	1.47	2.02	PCBM
	1.839	1.794	3.42	3.50	1.88	4.57	PffBT4T2OD *π*–*π* stacking (010)
In‐plane	0.295	0.294	21.30	21.38	21.93	17.51	PffBT4T2OD lamellar (100)
	N/A	0.583	N/A	10.77	N/A	12.59	PffBT4T2OD lamellar (200)
	N/A	0.641	N/A	9.80	N/A	1.59	PCBM
	1.390	1.353	4.52	4.64	2.67	3.53	PCBM

^a)^ Crystallite coherence length (CCL) calculated using Scherrer equation: $\mathrm{CCL}=\frac{\kappa \lambda }{\beta \;cos\;\theta }$,where:*κ* = dimensionless shape factor (0.94); *λ* = X‐ray wavelength (0.134 nm); *β* = FWHM of peak in radians; *θ* = Bragg angle in radians.

**Figure 5:**
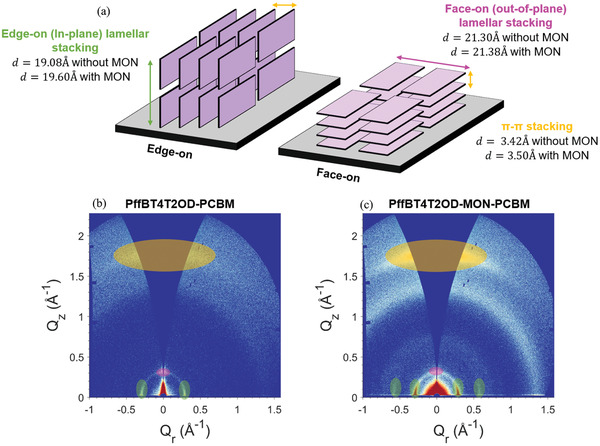
a) Illustration of edge‐on and face‐on PffBT4T2OD molecular orientations with the characteristic length scales extracted from GIWAXS measurements for b) PffBT4T2OD:PCBM and c) PffBT4T2OD:MONs:PCBM.

We evaluated the molecular organization in the out‐of‐plane direction and found that the *π*–*π* stacking (010) peak is shifted from 1.84 to 1.79 Å^−1^ upon incorporation for MONs and corresponds to an increase in the *π*–*π* stacking distance from 3.42 to 3.50 Å. We have determined the *π*–*π* crystal coherence length (CCL) from the full‐width half maximum of the *π*–*π* stacking (010) peak using the Scherrer equation. FWHM values are provided in Table S3, Supporting Information. We find that this also increases from 1.88 to 4.57 nm on incorporation of the MONs, indicating an increase in lattice ordering. The in‐plane GIWAXS patterns for reference PffBT4T2OD:PCBM films showed well‐defined peaks at 0.29 Å^−1^ assigned to lamellar stacking (100) and a weak *π*–*π* stacking (010) peak at 1.80 Å^−1^. As expected, PCBM is found to be largely amorphous with short range structural order visible as an isotropic ring at 1.4 Å^−1^ corresponding to the approximate size of PCBM molecules (4.5 Å). With the addition of MONs, the PffBT4T2OD:MON:PCBM films show the same in‐plane stacking peaks as observed in the PffBT4T2OD:PCBM films, with no shifts detected in their position. This indicates that the donor polymer and acceptor fullerene maintain their molecular structure in the ternary blend even upon the incorporation of the MONs. However, there is an increase in the intensity of the (100) polymer peak and an additional second order (200) polymer peak is visible at 0.59 Å^−1^ corresponding to enhanced lamellar stacking. This indicates that the incorporation of MONs drives an increase in long range molecular order of the polymer oriented face‐on to the substrate. With the addition of MONs, the enhanced intensities of the in‐plane lamellar stacking peak and out‐of‐plane *π*–*π* stacking peak are indicative of a higher degree of molecular order with PffBT4T2OD aligned in a favorable face‐on orientation (refer to the polymer packing illustration in **Figure** **5**).

**Figure 6:**
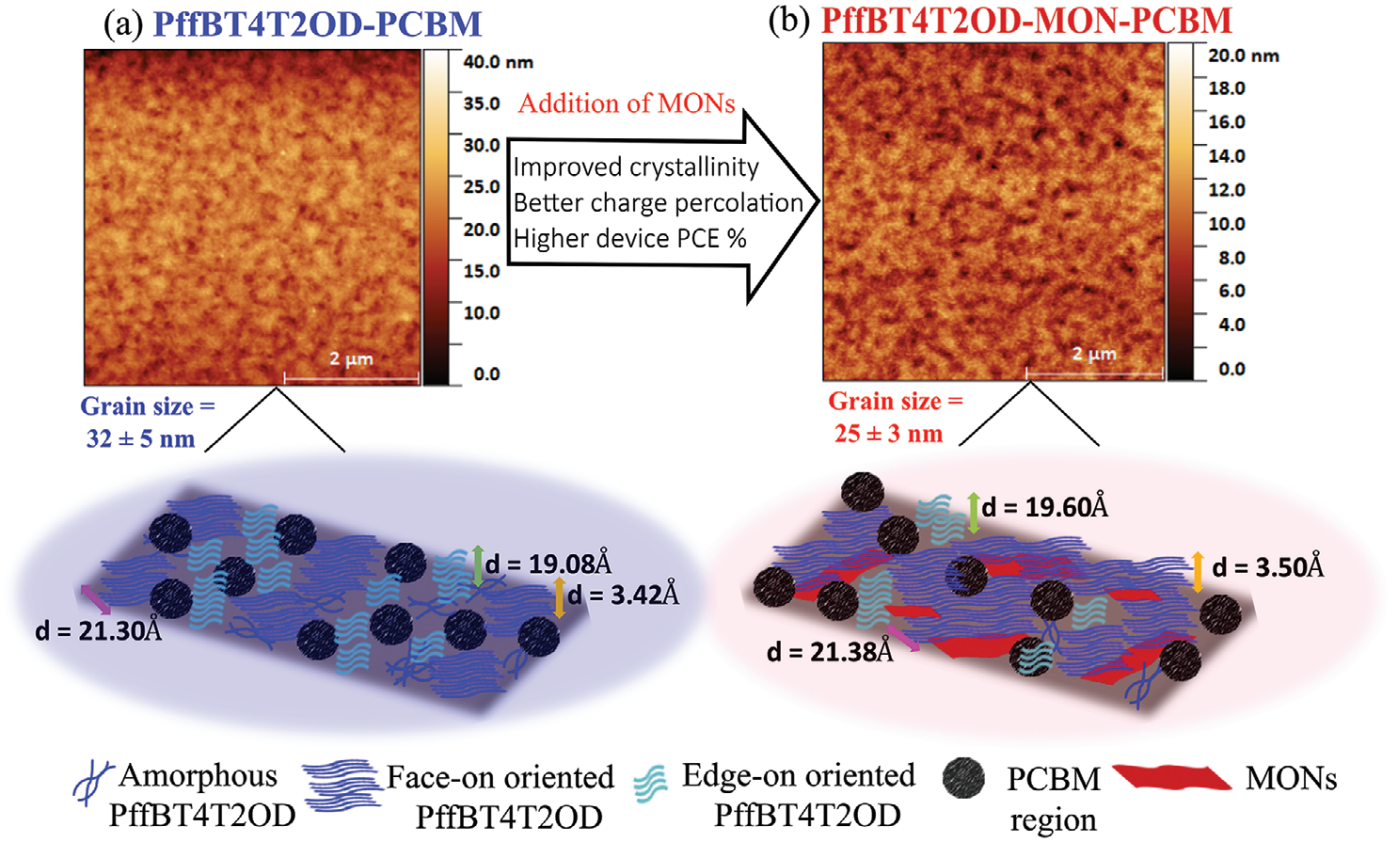
AFM images showing the morphology of the optimized device photoactive layers without (a) and with (b) addition of MONs. The corresponding cartoons below highlight the role of the nanosheets in promoting face‐on orientation of the PffBT4T2OD polymer, thereby resulting in improved crystallinity, better charge percolation pathways, and improved device PCE.

AFM imaging of the reference and MON based films was performed in order to characterize morphology, with a spatial Fourier transform function applied to extract the grain size. In the reference films, the characteristic length scale was found to be (32 ± 5) nm which reduced to (25 ± 3) nm upon incorporation of the MONs. (**Figure** **6**) This shows that all films have domains that have a size favorable range for exciton dissociation.

The performance metrics of both PffBT4T2OD:PCBM devices and PffBT4T2OD:MON:PCBM devices stored outside the glovebox at room temperature for 9 months were re‐tested (Section S10, Figure S13, Supporting Information). The average PCE of the devices after 9 months was 4.7% with MONs compared to 3.6% for the devices without nanosheets. This indicates that the presence of the nanosheets has no detrimental effect on the stability of the devices over time and continues to offer enhanced performance compared to devices without nanosheets.

## 3 Discussion of the Role of MONs within the Devices

The ultrathin dimensions of MONs enable the incorporation of MOF type materials as ternary additives within the BHJ of OPV devices for the first time. Our initial choice to investigate zinc porphyrin MONs was based on their complimentary optical and electronic properties. The porphyrin units in the MONs have two sets of absorption peaks which are complimentary to many of the systems investigated, S‐absorption between 430 and 450 nm and Q‐absorption bands between 550 and 580 nm. Amorphous systems such as PCDTBT, PTB7‐Th, and PBDB‐T showed marginal increases in PCE of between 0.2% and 0.7%. We attribute this to complimentary absorption by the porphyrin unit as observed by UV–vis and marginal increases in *J*_SC_ values of upto 1 mA cm^−2^ in these systems. In PffBT4T2OD:PCBM systems the HOMO of the MONs is above that of the donor polymer and the complimentary absorption and emission spectra allow for the MONs to act as sensitizers to extend the absorption range. It is worth noting therefore that, even in the absence of any other effects, the intrinsic optical and electronic properties of the MONs allow 5–20% of the material in the BHJ to be replaced. The highly tunable structure of MONs provides a clear route to optimizing their properties in order to enhance OPV performance by increasing the range of solar spectrum that can be absorbed, providing alternative charge transport pathways or reducing costs in OPV systems.

In line with our initial hypothesis, the crystallinity of the donor polymer was the biggest determinant of the size of the increase in performance observed. Addition of MONs to semi‐crystalline polymer systems resulted in significant enhancements in PCE, more than doubling in the case of P3HT‐MON‐ICBA and leading to the highest reported PCE's for PffBT4T2OD:MONs:PCBM. In all of these semi‐crystalline systems we see underlying improvements in both *J*_SC_ and FF. These much more significant increases in performance point to an additional mechanisms being important in these systems which do not apply to the amorphous systems.

GIWAXS analysis showed that PffBT4T2OD peaks increased in intensity following the addition of MONs indicating an increase in the crystalline fraction, as observed previously for P3HT.^[^ ^37^ ^]^ Scherrer analysis shows that the *π*–*π* stacking crystal correlation length of the polymer increases from 1.88 to 4.57 nm after the addition of MONs. An additional insight from this GWAXS study is that the addition of MONs promotes face‐on orientation of the PffBT4T2OD chains, resulting in stronger *π*–*π* stacking out‐of‐plane and stronger lamellar stacking in‐plane. This is the preferred orientation for photovoltaic devices because charge transport takes place in the perpendicular direction. UV–vis studies of the thin films showed an increase in intensity of the peak at 700 nm which is associated with the increased fraction of more ordered, and therefore more highly conjugated, PffBT4T2OD polymer.^[^ ^57^, ^58^ ^]^ This is a favorable change as there are more ordered charge transport pathways within the active layer, leading to reduced non‐geminate recombinations and increased *J*_SC_. This observation, combined with AFM studies which showed a reduction in domain size following addition of the MONs explains increased hole mobilities observed. The increase in electron mobility is likely a result of improvements in the network formation of the fullerene phase which has been reported in other studies where increased phase separation in thin films resulting from improved crystallinity of the donor polymer.^[^ ^69^, ^70^ ^]^

These results therefore validate our initial hypothesis that ZnTCPP MONs can significantly enhance the performance of other semi‐crystalline OPV devices. Device morphology is the key to optimizing device performance and in addition to any intrinsic benefits they bring, the MONs can have an amplified effect by improving the morphology of the surrounding components. MONs align parallel to the surface and act as templates during the spin‐coating procedure, improving the crystallinity and orientation of the surrounding polymer and reducing domain size. This in turn results in increased absorption and charge transport and so significant improvements in PCE. The modular structure of the MONs allows for systematic investigations to identifying the surface interactions that lead to this templating effect and optimize them to enhance the absorption, transport properties, and stability of semi‐crystalline polymers in a wide range of devices.

## 4 Conclusion

We have demonstrated that porphyrin‐based MONs can be used as additives to enhance the performance of a variety of OPV devices. The favorable nanoscale size dimensions, anisotropic structure, and electronic band‐gap of the MONs allow them to be incorporated in high concentrations into the active layer of BHJ devices through a simple spin‐coating procedure. Devices based on amorphous polymer donor‐systems benefitted from the additional light‐absorption of the porphyrin units in MONs, which lead to small increases in the device *J*_SC_. However, semi‐crystalline polymer systems showed remarkable improvements in both *J*_SC_ and FF which resulted in substantially higher PCE, more than doubled in the case of P3HT:ICBA devices. Most remarkably, a PCE of 12.3% was achieved in a PffBT4T2OD:MON:PCBM device, which—to the best of our knowledge—is the highest reported fullerene‐based device.

GIWAXS, AFM, and dark injection studies show that the MONs also improve polymer crystallinity and prevent PCBM aggregation. We suspect that this generates enhanced charge percolation pathways and enable better phase separation between PffBT4T2OD and PCBM, leading to the high‐power conversion efficiencies.

These results establish the templating effect of MONs as a general approach to improve the performance of semi‐crystalline polymer‐based devices. We believe that it should also be possible to engineer MONs that act as electron donors in fullerene‐free OPVs. Enhanced ordering and crystallinity are also desirable properties in other polymer‐electronic applications such as OLEDs and OFETs, and thus MONs hold significant potential as additives to create a new generation of organic electronic devices having improved performance.

## Conflict of Interest

D.G.L. is the co‐founder and director of the company Ossila Ltd that retail materials and equipment used in the fabrication and evaluation of organic electronic devices including thin‐film photovoltaics. All other authors declare no conflict of interest.

## Author Contributions

K.S. carried out the synthesis and characterization of MONs, device fabrication, testing, and related analysis and drafted the manuscript. R.C.K. carried out the GIWAXS studies and related data analysis. E.L.K.S. carried out the EQE measurements. J.C., A.I., and D.G.L. supported the experimental design, data analysis, and aided in the drafting of the manuscript. J.A.F. designed and coordinated the project and helped in drafting the manuscript.

## Supporting information

Supporting Information

Supporting Information

Supporting Information

## Data Availability

The data that support the findings of this study are available in the supplementary material of this article.
