# Supplementary material for: Metal‐Organic Framework Nanosheets as Templates to Enhance Performance in Semi‐Crystalline Organic Photovoltaic Cells
Source: Adv Sci (Weinh). 2022 May 22;9(21):2200366. doi: 10.1002/advs.202200366 (PMC9313490; doi:10.1002/advs.202200366)
Supplement: Supplementary file 1 — Supporting Information [file ADVS-9-2200366-s003.pdf]

Metal-organic framework nanosheets as templates to enhance performance in semi-crystalline organic photovoltaic cells

Kezia Sasitharan, Rachel C. Kilbride, Emma L.K. Spooner, Jenny Clark, Ahmed Iraqi, David G. Lidzey and Jonathan A. Foster

Supplementary information

Table of contents

1. General experimental procedures

2. Syntheses

3. Fabrication of the photovoltaic devices

4. Statistical analysis

5. EQE

6. GIWAXS analysis

7. AFM imaging and grain size analysis

8. SCLC devices for charge carrier mobility measurements

10. References

1. General experimental procedures

Commercial solvents and reagents were used without further purification. Synthesis of organic ligands was carried out in dry glassware with a nitrogen overpressure. Solvothermal synthesis of metal-organic frameworks was undertaken using borosilicate vials with Teflon faced rubber lined caps.

NMR spectra were recorded on a Bruker Advance DPX 400 spectrometer. Chemical shifts for ^{1}H are reported in ppm on the δ scale; ^{1}H chemical shifts were referenced to the residual solvent peak. All coupling constants are reported in Hz. Mass spectra were collected using a Bruker Reflex III MALDI-TOF spectrometer. Elemental analyses were obtained on a vario MICRO CHNS elemental analyzer equipped with a thermal conductivity detector. X-Ray powder diffraction patterns were collected using a Bruker D8 Advance powder diffractometer equipped with a copper K_α source (λ=1.5418 Å) operating at 40 kV and 40 mA. The instrument was fitted with an energy-dispersive LYNXEYE detector. Measurements were carried out using a fixed goniometer stage with a rotating flat plate sample holder. IR

spectroscopy was performed on a Perkin-Elmer Pyris TGA from 30-600°C at 10 °C min^{-1}, under a 20 mL min^{-1} nitrogen flow. UV-Vis absorption spectra were collected on a Cary 5000 UV-Vis-NIR instrument. PL spectra were recorded using a Horiba FluoroMax spectrofluorometer using 500 nm excitation. The fluorescence emission was corrected for sample absorption at the excitation wavelength to produce an emission spectrum proportional to the fluorescence quantum efficiency.

Nanoscopic characterisation was performed using a Bruker Multimode 5 AFM, operating in soft-tapping mode under ambient conditions. Bruker OTESPA-R3 cantilever were used, with a drive amplitude and nominal resonance frequency of 20.4 mV and 290 kHz, respectively. Images were processed using standard techniques with the free Gwyddion software.

2. Syntheses

a. Synthesis of *meso-tetracarboxyphenyl porphyrin* (TCPP) ligand

10 mL of pyrrole (98%, light brown solution) was distilled under vacuum at 80°C to give 5 mL of a clear solution. 4-formylbenzoic acid (3.0 g, 20 mmol) was dissolved in propionic acid (100 mL) and freshly distilled pyrrole (1.4 mL, 20 mmol) was added by syringe. The solution immediately darkened and was refluxed for 15 hours. The mixture was chilled in a fridge for 10 hours before collection of the solid by vacuum filtration. The solid was washed with hot water (2 x 20 mL) and dried under vacuum to give the product as a black powder (3.23 g, 4.0 mmol, 80 %). Elemental Analysis calculated for $C_{48}H_{30}N_4O_8$: Expected: C, 72.91; H, 3.82; N, 7.09. Found: C, 72.95; H, 4.08; N, 6.82

λ_{max} (nm): 420 (π-π^*)

^{1}H-NMR (d_6-DMSO) δ/ppm: 8.85 (8H, s, β-pyrrolic H), 8.38 (8H, d, *J* = 8.0, Ar-H), 8.32 (8H, d, *J* = 8.0, Ar-H), -2.95 (2H, s, internal pyrrole NH).

MALDI-TOF: m/z 791.2 ($[MH]^+$)

b. Synthesis of bulk Zn_2(Zn-TCPP)(DMF)

H_2TCPP (7.9 mg, 0.03 mmol), $Zn(NO_3)_2.3H_2O$ (8.9 mg, 0.09 mmol), DMF (1.5 mL) and ethanol (0.5 mL) were mixed at room temperature and heated at 80°C for 24 hours. Purple crystals were collected by centrifugation at 4500 RPM for 10 minutes and repeatedly washed in ethanol until the supernatant became clear (Yield 87%)

Elemental analysis $C_{48}H_{24}N_4O_8Zn_3(H_2O)_4(DMF)_3$: Expected C, 53.57; H, 4.50; N, 8.33 Found C, 52.76; H, 4.77; N, 8.88.

A slight excess of Zn around the edge of the MOF and possible nitrate counter ions could be the reason for lower carbon content and higher nitrogen.

c. Exfoliation of MOF into MONs

5 mg of Zn_2(ZnTCPP) MOF was added to a 12 mL glass vial along with 6 mL of the desired solvent. The sample was mixed in a vortex mixer for 30 seconds to disperse the sediment. The samples were sonicated using a Fisherbrand Elmasonic P 30H ultrasonic bath (2.75 L, 380/350 W, UNSPSC 42281712) filled with water. Samples were sonicated for 60 min at a frequency of 80 kHz with 100% power and the temperature was thermostatically maintained at 16-20°C using a steel cooling coil. Sonication was applied using a sweep mode and samples were rotated through the water using an overhead stirrer to minimise variation due to ultrasound “hot-spots”. Following sonication, the vials were transferred to centrifuge tubes and centrifuged at 1500 RPM for 10 minutes to remove non-exfoliated particles.

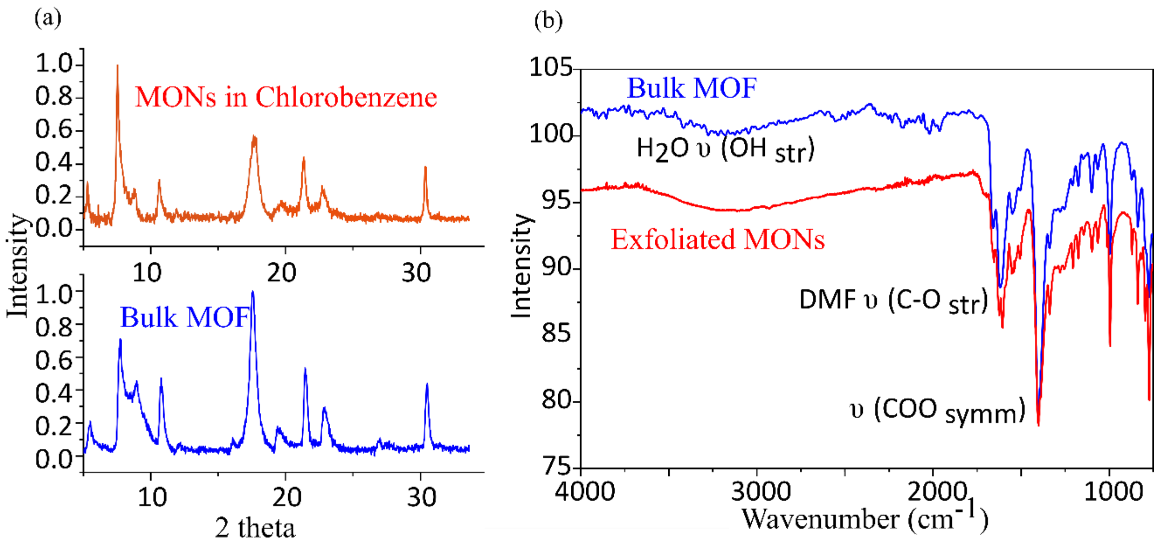

Figure S1 (a) Powder diffraction pattern for as-synthesized Zn_2(ZnTCPP) MON in comparison with the synthesized bulk MOF. The diffraction patterns are in accordance with the previous reports[51] (b) FTIR spectra of Zn_2(ZnTCPP) bulk MOF in comparison with exfoliated MONs.

3. Fabrication of the photovoltaic devices

a. Preparation of substrates

Pre-patterned ITO Glass substrates (20 mm x 15 mm) of 20 ohm/square resistance (Ossila) were cleaned via hellmenex (1-3vol%) in boiling DI water (10-minute sonication), followed by further washing in boiling DI water (10-minute sonication) and finally 5-10 minutes sonication in isopropyl alcohol**.** The substrates were dried with a nitrogen gun and placed in a UV-Ozone cleaner for 10 minutes to ensure removal of any surface particulates.

b. Deposition of ZnO

The precursor gel used to fabricate the ZnO electron transport layer was prepared by dissolving 0.2 g of zinc acetate dihydrate in 2ml 2-methoxyethanol with 55ul ethanolamine as stabilizer. The solution was then stirred in air for 12 hours to form a transparent gel which was spin-coated at 3000 rpm onto the ITO surface. After being baked at 200°C for 60 min in air, the ZnO coated substrates were transferred into the N2 filled glovebox.

c. Active layer deposition

i. P3HT-ICBA

P3HT (Sigma Aldrich) and ICBA (99 % purity, supplied by Ossila) were used as received. P3HT (10 mg/mL) was dissolved in chlorobenzene (CB) solvent. After heating at 60 °C for 10 minutes followed by cooling towards room temperature, the solution was filtered through

a 0.45 um PTFE filter. The P3HT solution in chlorobenzene was then mixed with a given wt % of Zn_2(ZnTCPP) MONs (5 % -20 % tested for optimal concentration) and stirred at 60 °C for an hour. Then 10 mg of PCBM was added and the final P3HT: Zn_2(ZnTCPP): ICBA solution was heated at 70 °C for an hour and cooled to room temperature prior to spin coating. The solutions were then spin-cast onto ITO/ZnO at 1000 rpm under a nitrogen atmosphere in a glove box, forming films of ∼150 nm as determined by dektak. For control devices, 1:1(wt/wt) (P3HT: ICBA) were spin cast at 2000 rpm in the same method as above to give films of ~150 nm. Solvent-vapour annealing of the films was carried out by placing the coated substrates in a sealed metal container containing 20 uL of solvent chlorobenzene) for 5 minutes.

ii. PCDTBT-PC71BM

PCDTBT (Ossila) and PCBM (99% purity, supplied by Ossila) were used as received. PCDTBT (4 mg/mL) was dissolved in chlorobenzene (CB) solvent. After heating at 60 °C for 10 minutes followed by cooling towards room temperature, the solution was filtered through a 0.45 um PTFE filter. The PCDTBT solution in chlorobenzene was then mixed with 5 mg Zn_2(ZnTCPP) MONs (1:0.5 wt/wt ratio of PCDTBT: Zn_2(ZnTCPP)) and stirred at 60 °C overnight. Then 16 mg of PCBM was added and the final PCDTBT: Zn_2(ZnTCPP): PCBM (1:0.5:4 wt/wt ratio) was heated at 70 °C for an hour and cooled to room temperature prior to spin coating. The solutions were then spin-cast onto ITO/ZnO at 700 rpm under a nitrogen atmosphere in a glove box, forming films of ∼100 nm as determined by dektak. For control devices, 1:4(wt/wt) (PCDTBT: PCBM) were spin cast at 700 rpm in the same method as above to give films of ~90 nm.

iii. PTB7Th-PC71BM

PTB7Th (Ossila) and PCBM (99% purity, supplied by Ossila) were used as received. The solutions were made at 35 mg/ml with a PTB7Th:PCBM ratio of (1:1.5). The PTB7Th-PCBM solutions were made in chlorobenzene and set at 60 °C for 10 minutes for dissolution. This was followed by cooling towards room temperature. Half of this solution was retained for the reference devices, and to the remaining PTB7Th solution in chlorobenzene, 5% by weight of MONs was added. The solutions were spin-cast at 2700 rpm for the reference devices and 3000 rpm for the MON devices, resulting in an active layer thickness of 120nm in both the cases.

iv. PBDBT-PC71BM

PBDBT (Ossila) and PCBM (99% purity, supplied by Ossila) were used as received. PBDBT:PCBM (20mg/mL) was dissolved in chlorobenzene (CB) solvent and set at 80°C overnight for dissolution. This was followed by cooling towards room temperature. And the solutions were ready for the reference devices. To another solution prepared in the same way, 5% by weight of MONs were added. The solutions were then spin-cast onto ITO/ZnO at 1000 rpm under a nitrogen atmosphere in a glove box, forming films of ∼150 nm as determined by dektak. For the reference devices the speed of spin cast was 700 rpm to give films of ~150nm.

v. PffBT4T2OD-PC71BM (also known as PCE11-PCBM)

To coat the active layer, for the reference devices we prepared an ink consisting of 1:1.2 wt ratio PffBT4T2OD: PCBM dissolved at 9mg/ml in chlorobenzene. The solvent also contained 3 % volume of 1,8-diiodooctane (DIO). The optimised MON based devices had 10% by weight of MONs added to the ink. The inks were stirred at 130℃ for 6 hours before spin-coating to prevent polymer aggregation. The inks were spin-coated at 1000 rpm onto ZnO/ITO substrates held at 110 ℃. This heating of the substrates was found essential for uniform coating of film. Following annealing at 110℃ for 10 minutes, the top electrode consisting of 10 nm of molybdenum oxide film capped by 100 nm Ag was thermal evaporated onto the active layer through a shadow mask. Finally, the devices were encapsulated using UV cured epoxy glue and a glass cover slip.

d. Top contact deposition and encapsulation

The ITO/ZnO/Active layer substrates were placed under a vacuum of <2E-6 mBar before thermally evaporating MoO_3 (5nm) and Silver (100nm) layers using a shadow mask. The devices were encapsulated using an epoxy resin (Ossila, E131).

e. Device testing

Device performance was determined under ambient conditions by measuring *J–V* curves using a Newport 92251A-1000 solar simulator, with devices illuminated through a 0.0256 cm^2 aperture mask. Before each set of measurements, the intensity was calibrated to 100 mW cm^{-2} using an NREL certified silicon reference cell. The applied bias was swept from 0.0 to +1.2 V and back again at a scan speed of 0.4 V s^{-1} using a Keithley 237 source measure unit.

4. **PffBT4T2OD: PCBM statistical analysis**

Literature PffBT4T2OD devices – These results were obtained by using the keyword 'PffBT4T-2OD' in scifinder. The search yielded 126 research items. All these records were examined and the following results included in the box plot shown in Figure3b main text, with the aim to compare and investigate how highly performing our devices were with respect to the ones reported so far.

Table S1 Literature reports on PCE-11 based OPV devices

Jsc (-mA/cm^2)	Voc (V)	FF (%)	PCE (%)	Ref
12.7	1	60	8.2	[1]
18.41	0.78	68.70	9.84	[2]
16.41	0.72	71.2	8.41	[3]
22.95	0.73	60	10.07	[4]
18.4	0.77	74	10.50	[5]
20.8	0.72	69.3	10.4	[6]
18.3	0.76	64	8.9	[7]
20.29	0.74	68.9	10.33	[8]
13.0	1.07	63	8.7	[9]
17.04	0.74	67	8.56	[10]
11.29	0.93	50	5.25	[11]
17.79	0.78	65.3	9.06	[12]
18.08	0.77	68.9	9.58	[13]
17.58	0.86	67.2	10.18	[14]
21.92	0.73	0.60	9.50	[15]
17.5	0.75	72.4	9.2	[16]
9.6	0.78	47.7	3.59	[17]
19.44	0.74	67.25	9.63	[18]
16.31	0.75	67	8.20	[19]
18.5	0.72	73	9.6	[20]
11.50	0.93	43	4.61	[21]
10.92	0.81	38	4.23	[22]
17.9	0.80	68.4	9.79	[23]

16.82	0.76	76.4	10.19	[24]
13.89	0.75	58.85	6.11	[25]
16.61	0.79	62.5	8.25	[26]
20.76	0.76	61.8	9.8	[27]
13.6	1.04	66.1	9.4	[28]
15.50	0.82	71	9.02	[29]
19.54	0.73	74.6	10.7	[30]
17.3	0.76	70	9.31	[31]
18.61	0.75	72.6	10.13	[32]
15.53	0.74	69	7.97	[33]
20.12	0.78	61.2	9.66	[34]
20.07	0.78	69	10.36	[35]
18.28	0.77	74.02	10.36	[36]
16.7	0.75	70	8.8	[37]
19.5	0.75	72.2	10.57	[38]
18.4	0.77	73.9	10.59	[39]
18.40	0.77	64	9.07	[40]
19.38	0.81	65	9.80	[41]
18.6	0.74	68	10.1	[42]
14.65	1.08	60	9.5	[43]
18.19	0.76	66.6	9.07	[43]
18.8	0.76	70	10.1	[44]
18.87	0.80	68.21	10.30	[45]
19.02	0.77	72.62	10.72	[46]
17.49	0.74	67.5	8.62	[47]

5. External Quantum Efficiency measurements

External quantum efficiencies were measured using a white light source that was monochromated using a Spectral Products DK240 monochromator that was then imaged on the PSC active area. The intensity of the monochromated light was determined using a calibrated silicon photodiode having a known spectral response. The external quantum efficiency was measured in the spectral range 400-700 nm using an Xtralien X100 (Ossila) source measure unit to determine the PSC photocurrent. The EQE spectra were collected

approximately 9 weeks after the fabrication of the devices, hence the deterioration in Jsc values.

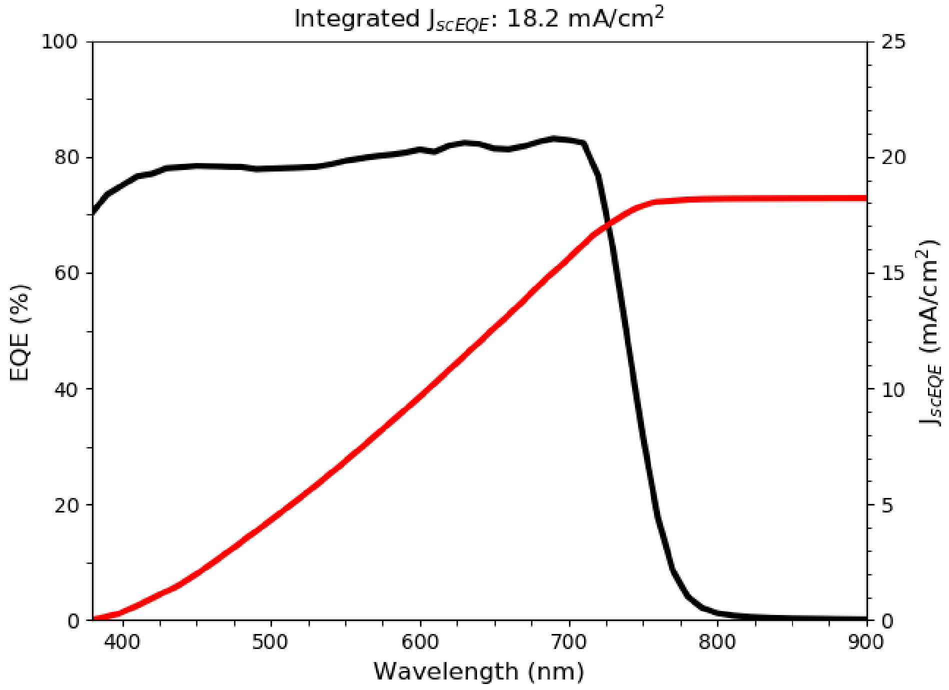

Figure S2. EQE of PffBT4T2OD-MON-PCBM devices with the integrated Jsc values

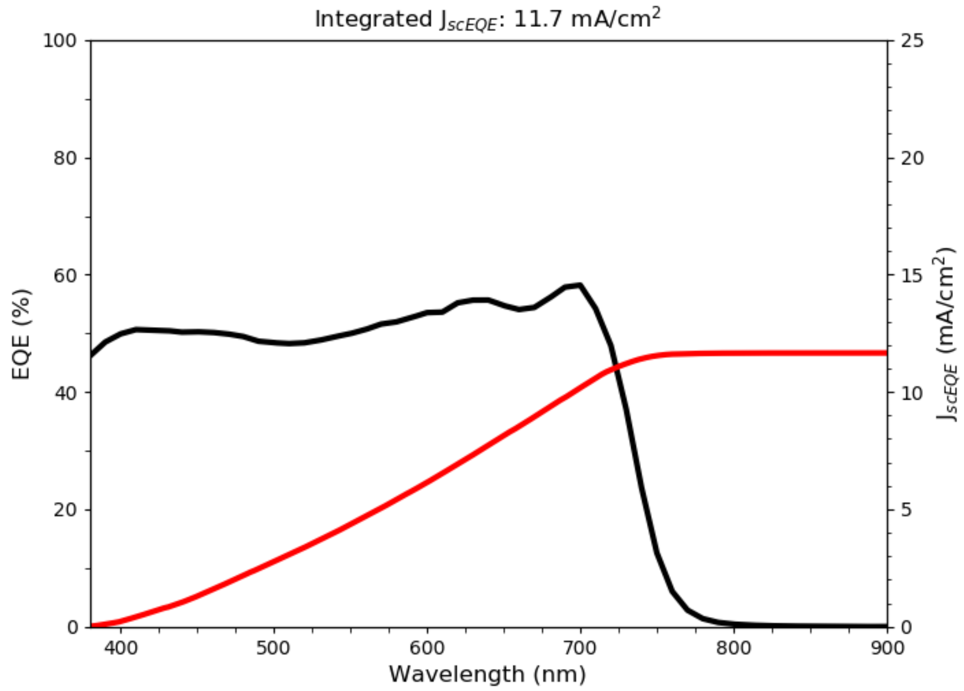

Figure S3. EQE of PffBT4T2OD-PCBM device with the integrated Jsc values

6. GIWAXS analysis

GIWAXS measurements were performed using a Xeuss 2.0 system (Xenocs, France) based at The Department of Chemistry, The University of Sheffield. The instrument is fitted with a MetalJet (Excillum, Sweden) liquid gallium X-ray source, providing a 9.24 keV X-ray beam collimated to a beam spot of 400 µm laterally at the sample position, measuring the full sample length. 2D GIWAXS patterns were acquired with a Pilatus3R 1M 2D detector (Dectris, Switzerland). The sample to detector distance (~307mm) was calibrated using a silver behenate calibrant standard in transmission geometry. Samples were measured in grazing incidence geometry using an incidence angle of 0.16° (near the critical angle, calculated to probe the entire film thickness) with the sample chamber and flight tubes held under vacuum to minimise the background scatter. The data was corrected, reshaped and reduced using the GIXSGUI MATLAB toolbox. [48] A guide displaying the areas of integration used to extract 1D intensity profiles from the 2D GIWAXS patterns is shown in Figure S7.

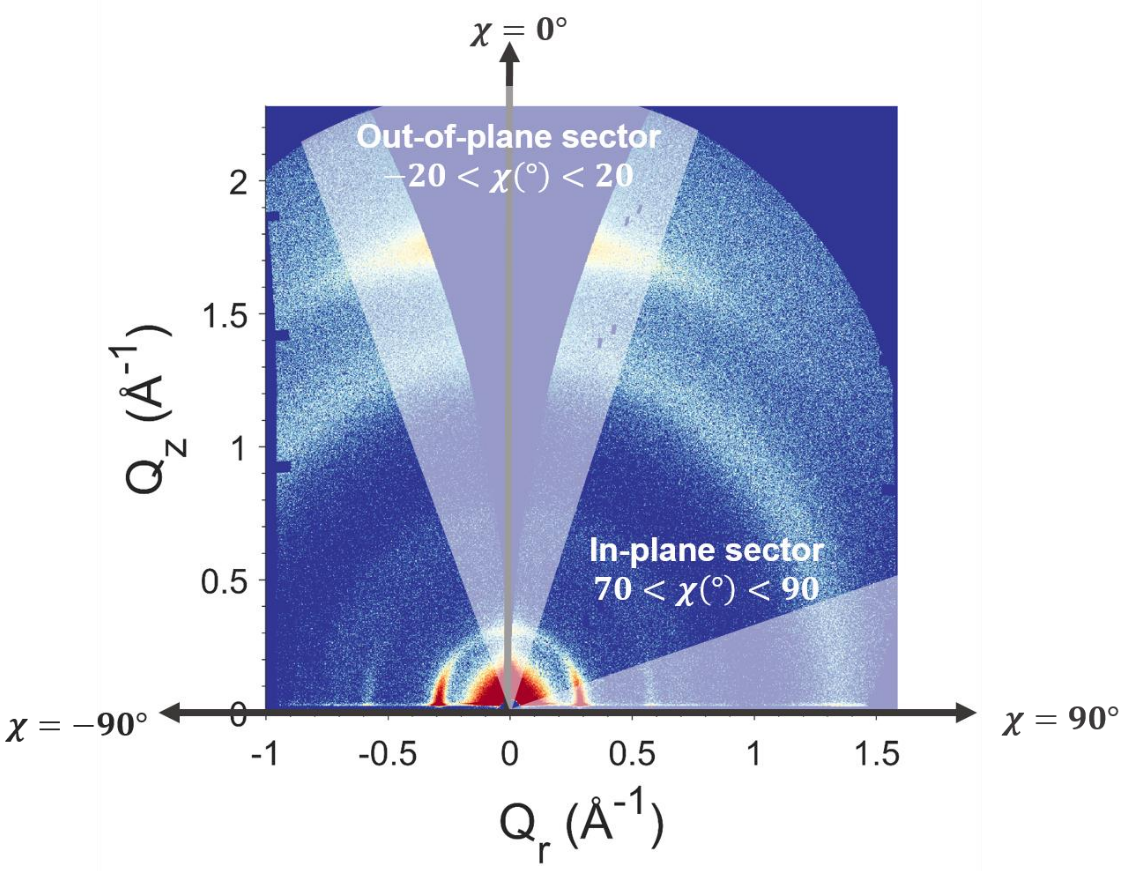

Figure S4 A guide showing the integration areas applied to the 2D GIWAXS patterns in this work to extract azimuthally integrated 1D Q-dependent intensity profiles. Data was integrated across the full Q range through various χ angles normal to the beam incidence at the detector; out-of-plane (in the Q_z direction, -20° < χ < 20°) and in-plane (70° < χ < 90°).

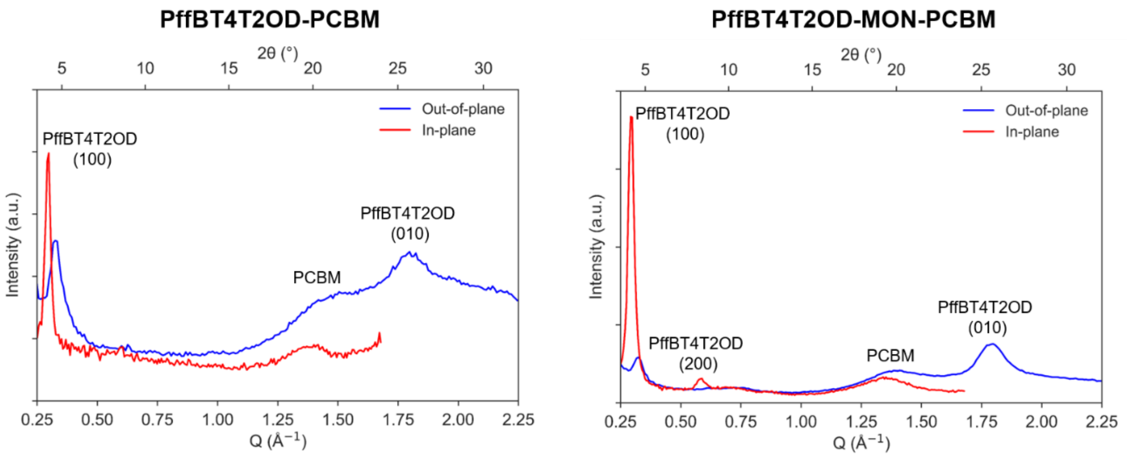

Figure S5 Azimuthally integrated 1D GIWAXS profiles in the in-plane and out-of-plane directions for PffBT4T2OD-PCBM and PffBT4T2OD-MON-PCBM.

**Direction**	**Q ($Å^{-1}$)**		**FWHM ($Å^{-1}$)**	
	**Without MON**	**With MON**	**Without MON**	**With MON**
**In-plane**	0.295	0.294	0.027	0.031
	N/A	0.583	N/A	0.047
	N/A	0.641	N/A	0.373
	1.390	1.353	0.224	0.169
**Out-of-plane**	0.328	0.321	0.092	0.073
	N/A	0.664	N/A	0.332
	1.484	1.416	0.406	0.295
	1.839	1.794	0.321	0.132

Table S2 Peak positions and full width half maximum (FWHM) values extracted from guassian curve fitting the scattering peaks in the out-of-plane and in-plane 1D intensity profiles for PffBT4T2OD-PCBM with and without MONs.

7. AFM imaging and grain size analysis

a. PffBT4T2OD-PCBM scan size = 5µm

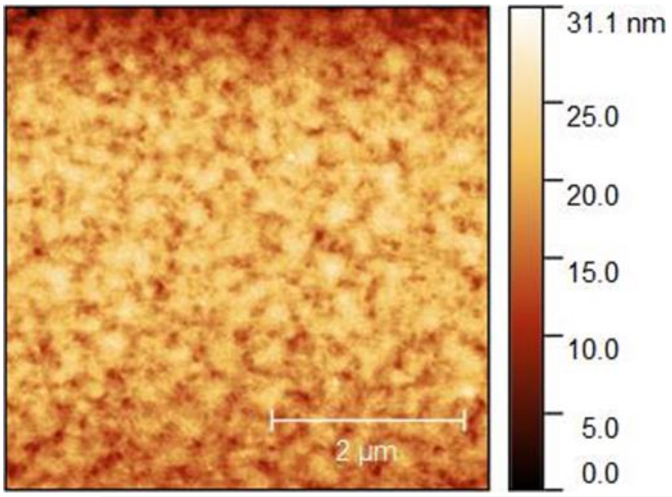

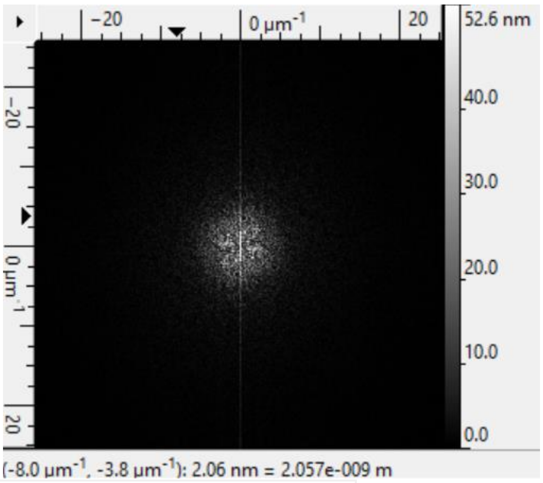

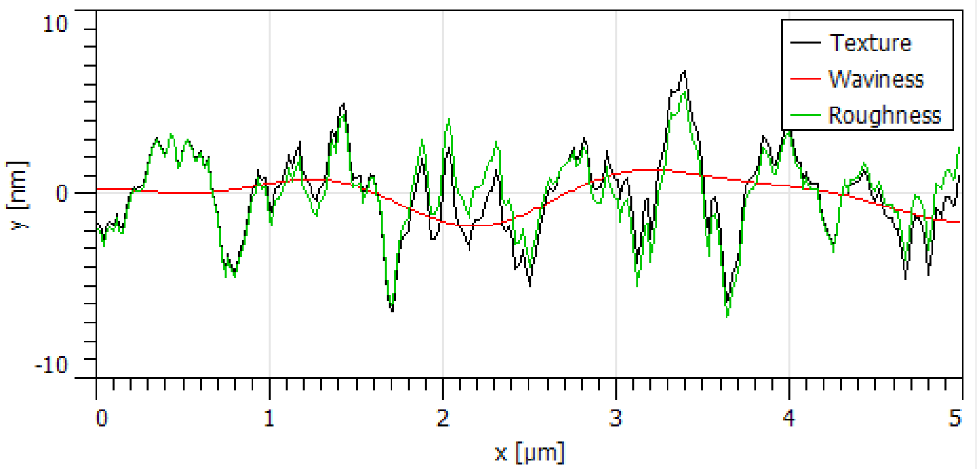

Figure S6 AFM grain size evaluation for a thin film of PffBT4T2OD-PCBM of scan size 5 µm

b. PffBT4T2OD-PCBM scan size = 2µm

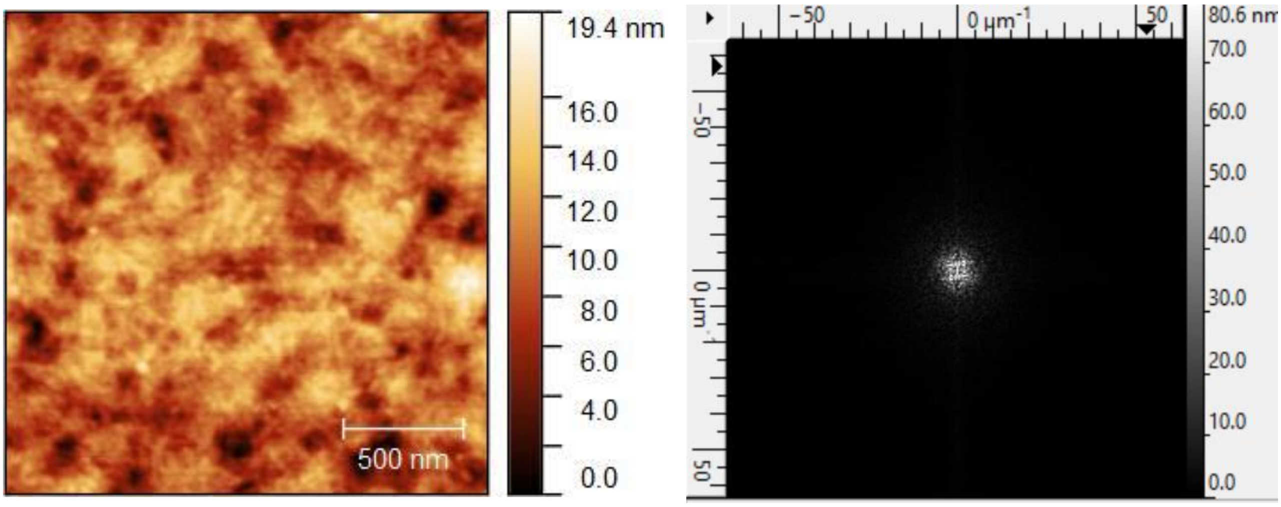

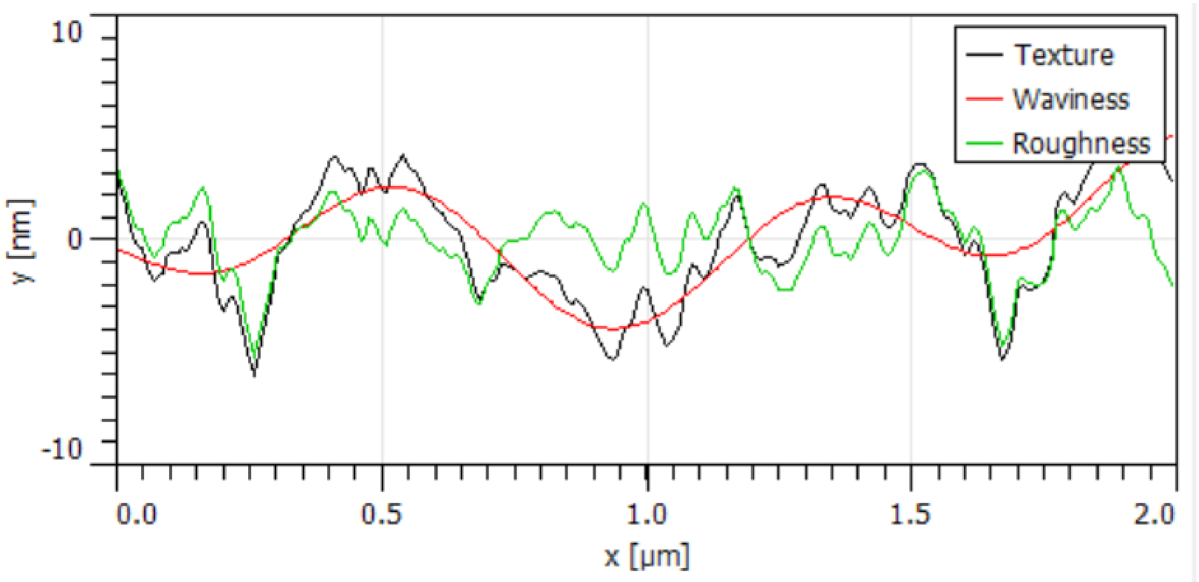

Figure S7 AFM grain size evaluation for a thin film of PffBT4T2OD-PCBM of scan size 2 µm

c. PffBT4T2OD-PCBM scan size = 500 nm

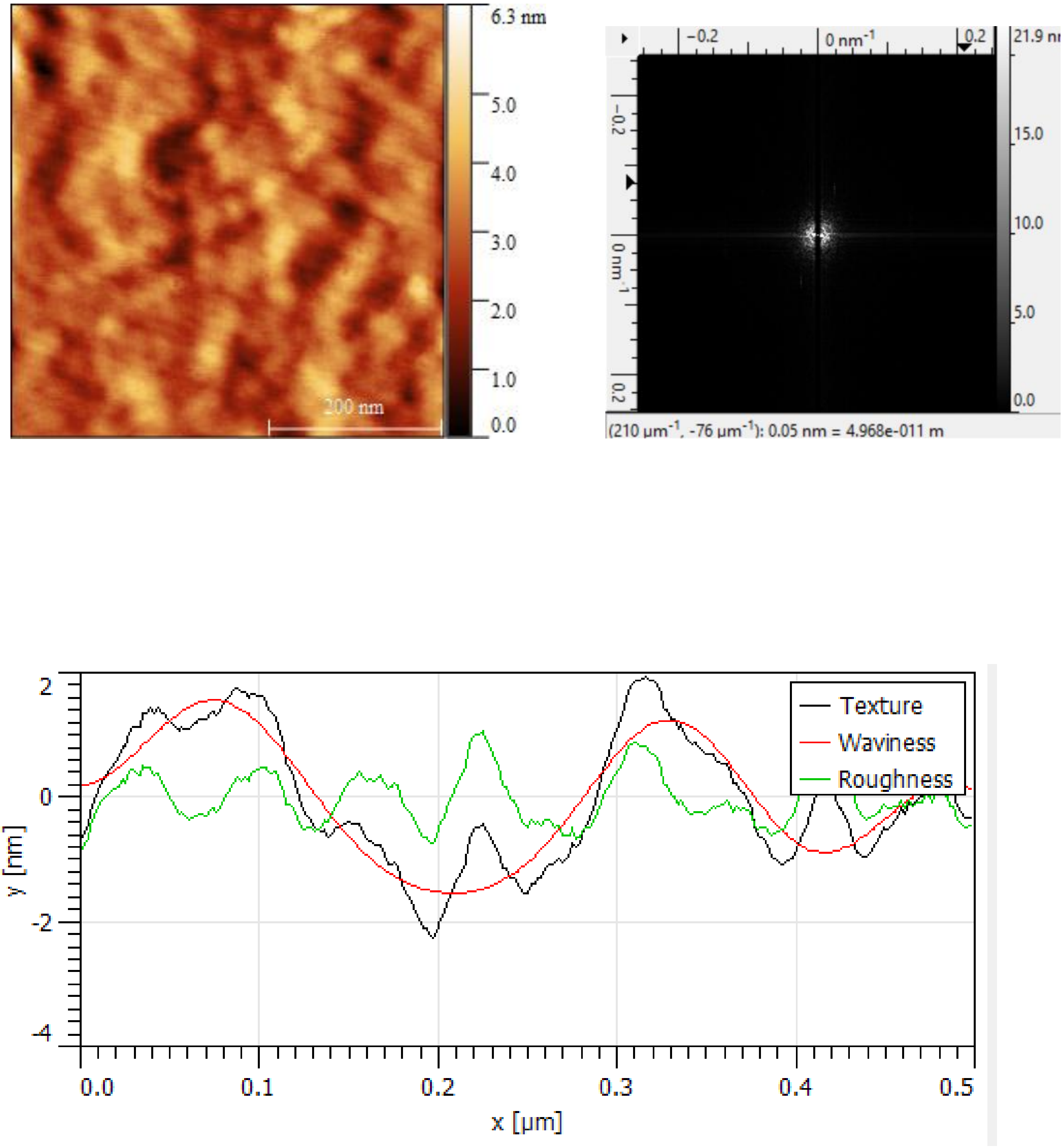

Figure S8 AFM grain size evaluation for a thin film of PffBT4T2OD-PCBM of scan size 500 nm

d. PffBT4T2OD-PCBM-MONs scan size = 5µm

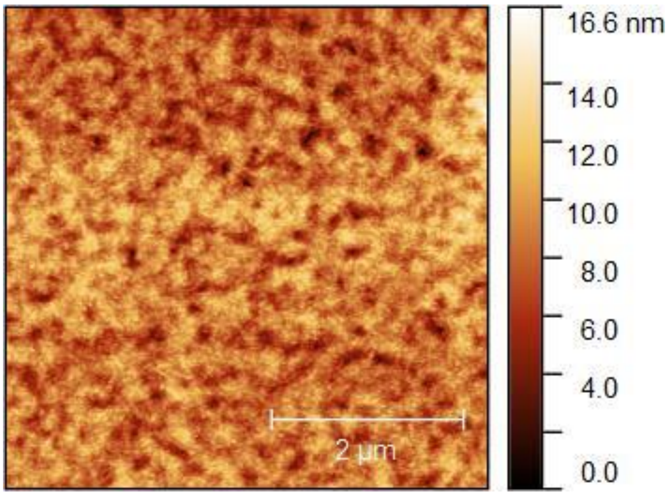

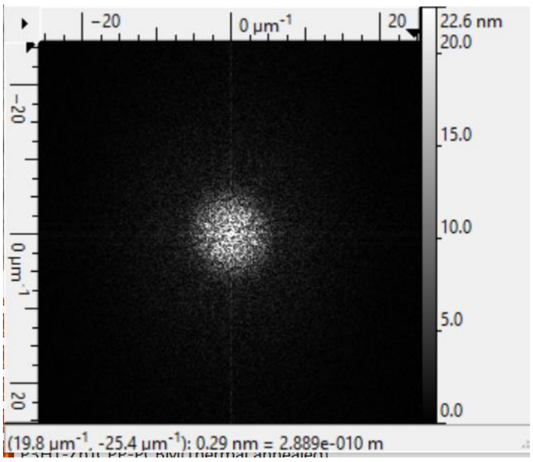

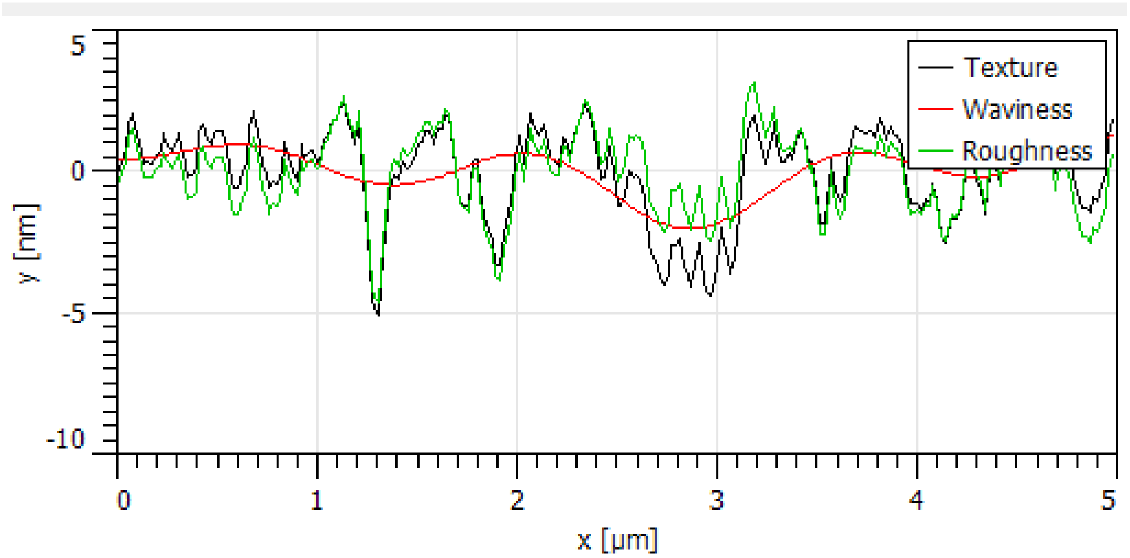

Figure S9 AFM grain size evaluation for a thin film of PffBT4T2OD-MON-PCBM of scan size 5 µm

e. PffBT4T2OD-PCBM-MONs scan size = 2μm

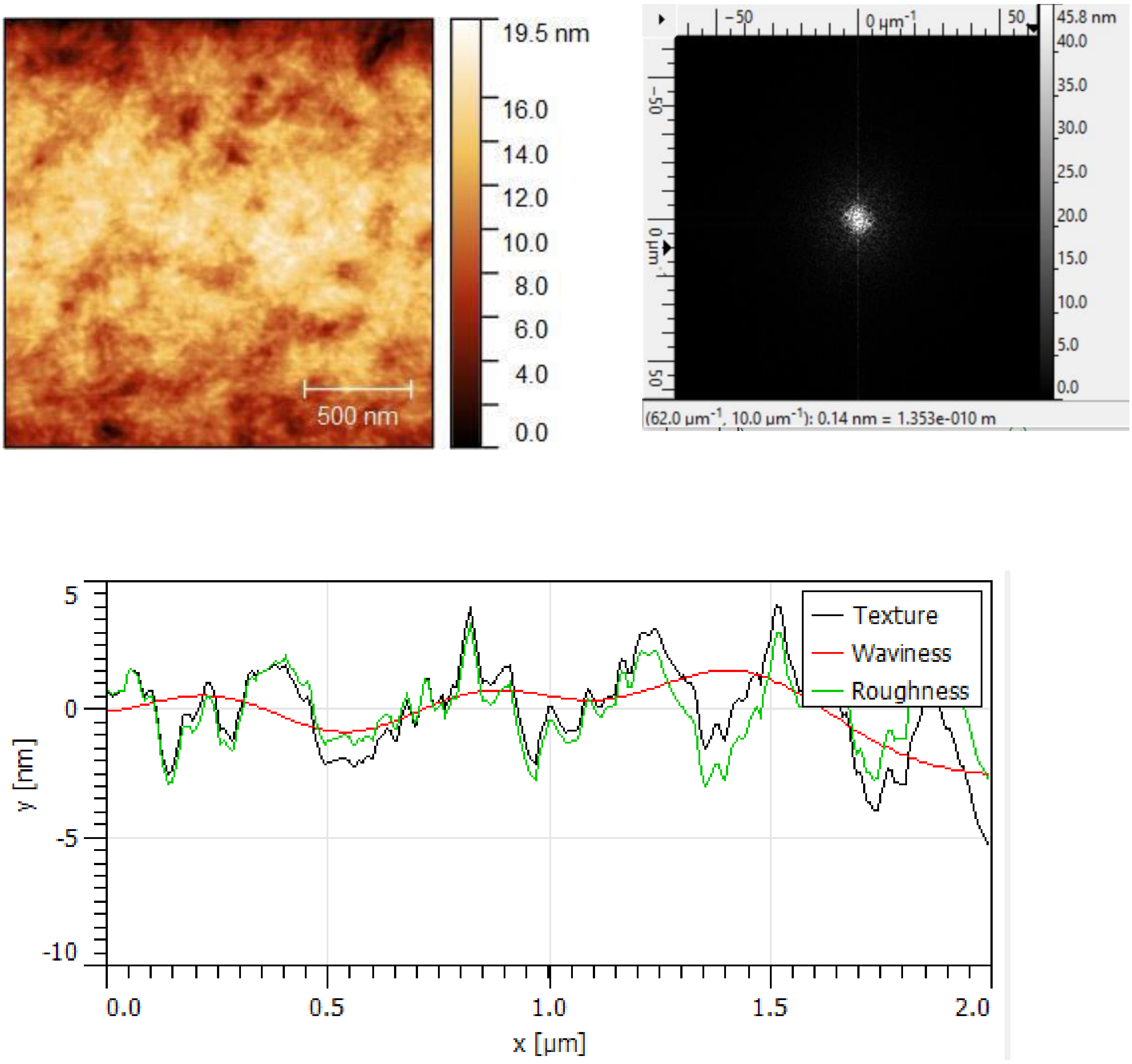

Figure S10 AFM grain size evaluation for a thin film of PffBT4T2OD-MON-PCBM of scan size 2 μm

f. PffBT4T2OD-PCBM-MONa scan size = 500nm

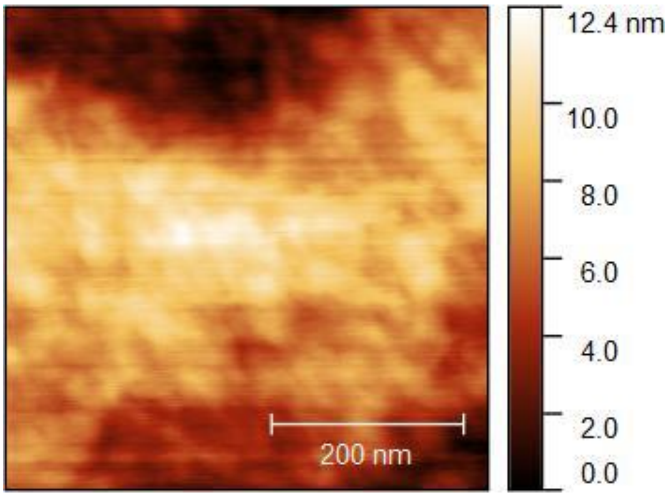

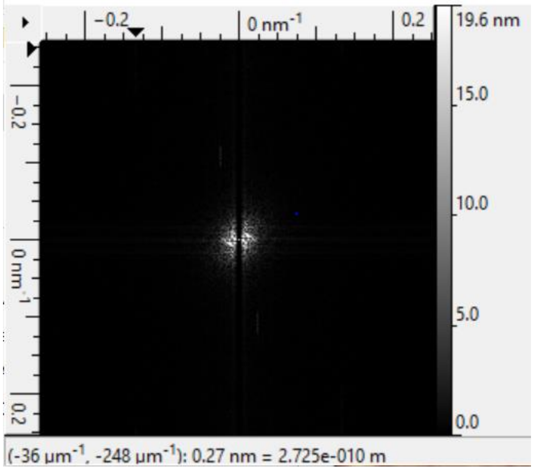

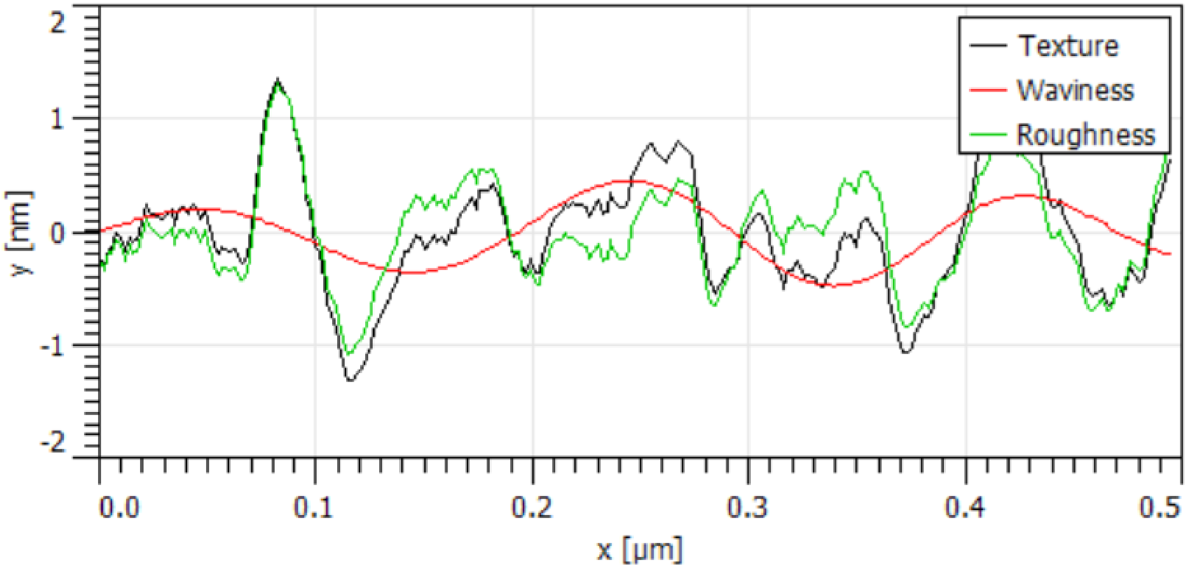

Figure S11 AFM Grain size evaluation for a thin film of PffBT4T2OD-MON-PCBM scan size 500 nm.

8. SCLC devices for charge carrier mobility measurement

The investigation of space-charge limited electron-only or hole-only devices for extraction of mobility values is a well-known and reliable method. Space-charge limited currents were tested in hole only devices with the configuration ITO:PEDOT/PSS:active layer:MoO_3:Ag and a well-established protocol from literature was used for data fitting and analysis[49]. The hole mobility was determined by fitting the dark current to the model of a single carrier SCLC current with field dependent mobility based on Mott-Gurney law. Electron only devices were fabricated with the configuration ITO:ZnO:Active layer:BCP:Ag to extract the electron mobility.

Table S3 Hole mobility and electron mobility extracted from SCLC holes only and electrons only devices for different thicknesses of active layer; Number of devices tested for each thickness=3.

**Device**	**Thickness**	**μh (Hole mobility)**	**μe (Electron mobility)**	**μh/μe**
PffBT4T2OD/PCBM	150 nm	1.35 X10 $^{-3}$	1.40 X10 $^{-3}$	0.96
PffBT4T2OD/MON/PCBM	150nm	2.07 X10 $^{-3}$	2.29 X10 $^{-3}$	0.90

Status: Complete

1) Raw data

Filename: PCE11-PCBM hole only

Selected data

Description of selected data: ITO/pedot:pss/PCE11-PCBM/MoO3/Ag

Film thickness, *d*	150	nm
Device area, *A*	0.025	cm^2

2)

Series resistance compensation?	TRUE	
Series resistance, *Rs*	20	Ω
Excluded voltages V_int >	1000000	V

3)

Built in voltage compensation?	TRUE	
V_BI value	-0.2	V
Exclude V_int <	0.00	V

4)

Relative dielectric constant, ε_r	3.5	
Internal voltage range selected for fitting		
min	0.01	
max	1	

5)

Fitted?	TRUE	
Fit results:		
μ0 =	1.13E-03	cm^2/Vs
γ =	1.00E-03	$cm^{1/2}/V^{1/2}$
Field range of good fit:		
min	1.97E+04	V/cm
max	6.81E+04	V/cm

**Mobility at selected electric field =**	**1.35E-03**	**cm^2/Vs**
at *F* =	3.00E+04	V/cm

Good fit at this electric field

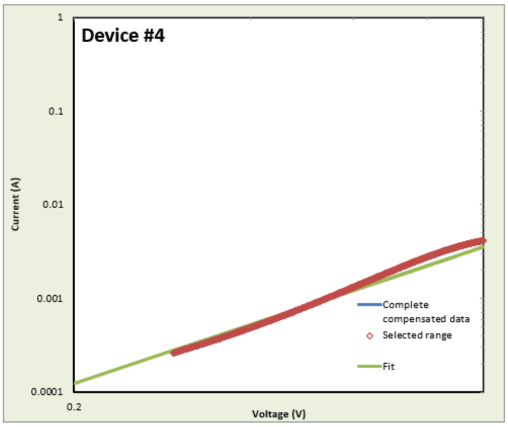

Status: Complete

1) Raw data

Filename: PCE11-Zn2(ZnTCPP)-PCBM hole only

Selected data

Description of selected data: ITO/PEDOT:PSS/PCE11-Zn2(ZnTCPP)-PCBM/MoO3/Ag

Film thickness, *d*	150	nm
Device area, *A*	0.025	cm^2

2)

Series resistance compensation?	TRUE	
Series resistance, *Rs*	10	Ω
Excluded voltages V_int >	1000000	V

3)

Built in voltage compensation?	TRUE	
V_BI value	-0.1	V
Exclude V_int <	0.00	V

4)

Relative dielectric constant, ε_r	3.5	
Internal voltage range selected for fitting		
min	0.01	
max	1	

5)

Fitted?	TRUE	
Fit results:		
μ0 =	1.27E-03	cm^2/Vs
γ =	2.82E-03	$cm^{1/2}/V^{1/2}$
Field range of good fit:		
min	1.77E+04	V/cm
max	6.24E+04	V/cm

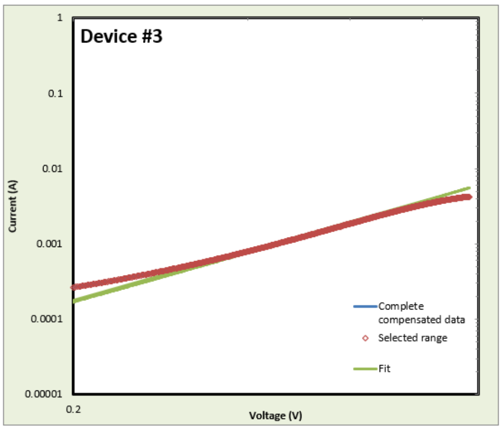

Status: Complete

1) Raw data

Filename: PCE11-Zn2(ZnTCPP)PCBM electron only

Selected data

Description of selected data: ITO/ZnO/PCE11-Zn2(ZnTCPP)PCBM/BCP/Ag

Film thickness, *d*	150	nm
Device area, *A*	0.025	cm^2

2)

Series resistance compensation?	TRUE	
Series resistance, *Rs*	20	Ω
Excluded voltages V_int >	1000000	V

3)

Built in voltage compensation?	TRUE	
V_BI value	-0.1	V
Exclude V_int <	0.00	V

4)

Relative dielectric constant, ε_r	3.5	
Internal voltage range selected for fitting		
min	0.01	
max	1	

5)

Fitted?	TRUE	
Fit results:		
μ0 =	1.79E-03	cm^2/Vs
γ =	1.40E-03	$cm^{1/2}/V^{1/2}$
Field range of good fit:		
min	1.40E+04	V/cm
max	6.15E+04	V/cm

**Mobility at selected electric field =**	**2.29E-03**	**cm^2/Vs**
at *F* =	3.00E+04	V/cm

Good fit at this electric field

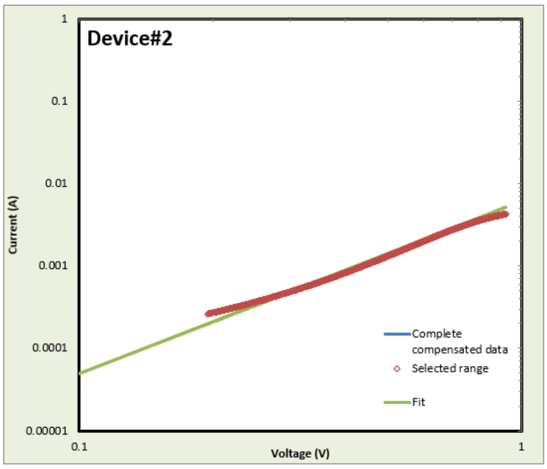

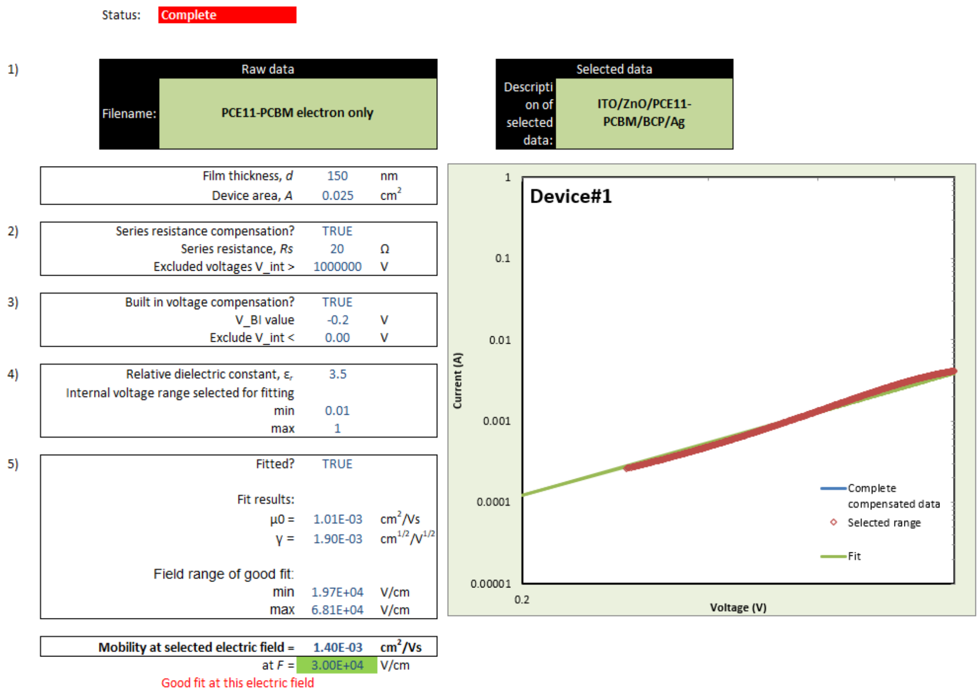

The devices were prepared following the same procedure described in the experimental section for photovoltaic devices, except that of the metal electrode. The mobilities were determined by fitting the dark current to the model of a single carrier SCLC current with field dependent mobility[49], which is described as

$$J= [(9\,\varepsilon_r\varepsilon_0\,\mu_0 V^2)/8L^3]\ \exp(\beta\sqrt{V}/L)$$

Where, J is the current, $\mu_0$is the zero-field mobility, ε_0 is the permittivity of free space, ε_r is the relative permittivity of the material, V is the effective voltage, and L is the thickness of the active layer.

9. Energy levels of PffBT4T2OD, MONs and PCBM

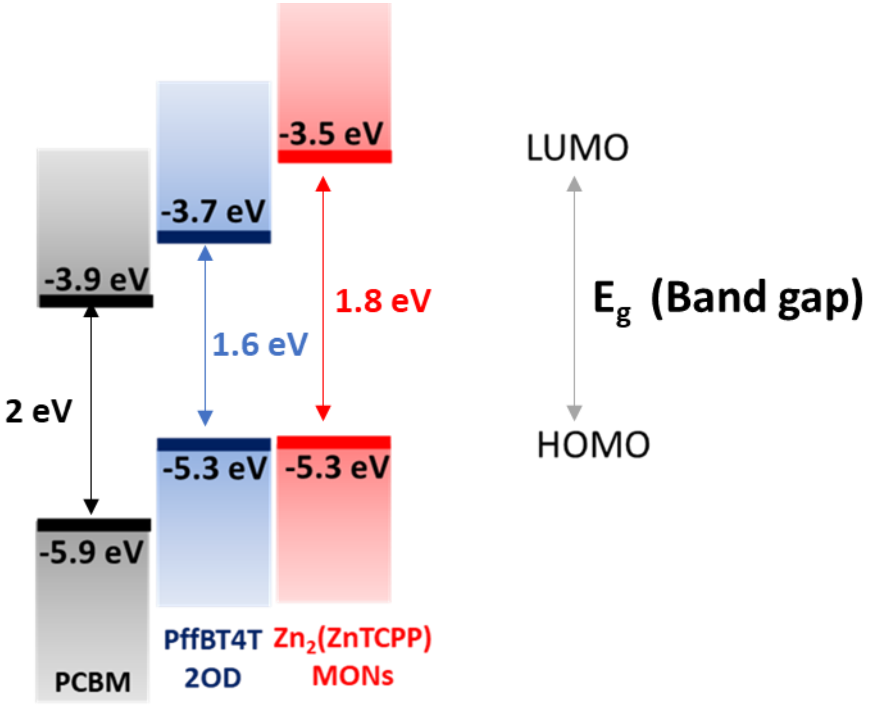

Figure S12. Representation of the HOMO-LUMO level alignment of the components in the ternary device – PffBT4T2OD: MON: PCBM

We have previously calculated the HOMO-LUMO energy levels of the MONs derived from the onset of oxidation and reduction in cyclic voltammetry.[50] Here, we use the calculated values for MONs to present the energy level diagram of the ternary components in the PffBT4T2OD-MON-PCBM device. The values for PffBT4T2OD and PCBM used here have been sourced from the supplier's website. The energy gap of MONs is larger than the donor polymer. In this case, we speculate that it can act as a sensitiser to extend the absorption range, and at the excited state it can transfer energy to the donor through Dexter or FRET mechanism. A pre-requisite for this energy transfer is that there should be significant overlap between the emission of the sensitizer (MON) and the absorption of the donor to allow efficient energy transfer. The MONs show a broad PL emission between 500-800 nm,[50] and PffBT4T2OD absorbs between 400-750 nm with an absorption maxima at 700 nm (Figure 3c, main text). Therefore, there is a significant overlap between the emission of MONs and absorption of the donor polymer to facilitate efficient energy transfer.

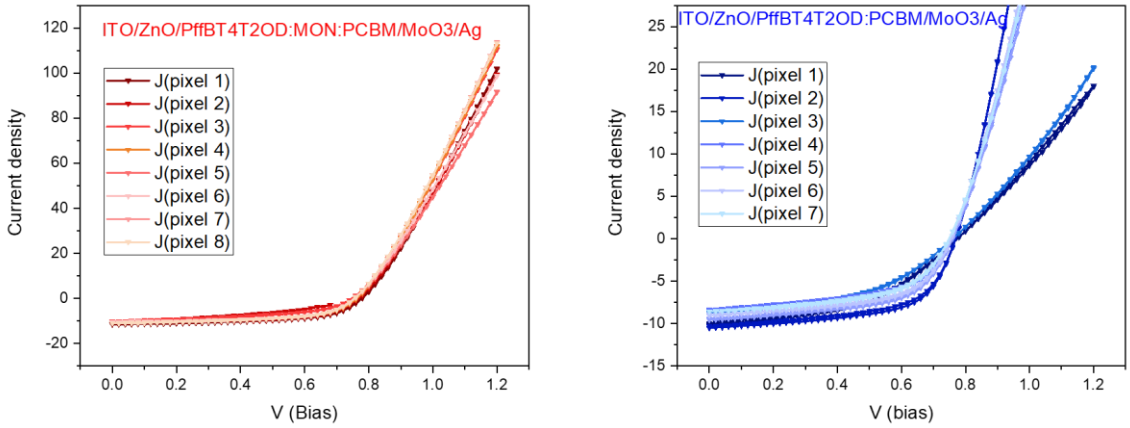

10. Device stability

Figure S13. J-V Curves of PffBT4T2OD:MON:PCBM and PffBT4T2OD:PCBM device stored in air, room temperature for 9 months.

Averaged over the 8 pixels, the PCE values are :

PffBT4T2OD:MON:PCBM = 4.7%

PffBT2OD:PCBM = 3.6%

PffBT4T2OD-MON-PCBM

Parameter	Pixel 1	Pixel 2	Pixel 3	Pixel 4	Pixel 5	Pixel 6	Pixel 7	Pixel 8
Voc (V)	0.77868	0.76793	0.74831	0.7625	0.756036	0.754435	0.756939	0.755823
Jsc (mA/cr	-11.5234	-11.0352	-10.25	-10.5898	-10.7891	-10.5547	-11.0312	-11.0391
FF (%)	59.2441	59.4634	48.7399	59.7058	59.3342	60.0447	58.4676	57.9225
Vmpp (V)	0.62	0.6	0.56	0.6	0.6	0.6	0.6	0.6
Jmpp (mA	-8.57422	-8.39844	-6.67578	-8.03516	-8.06641	-7.96875	-8.13672	-8.05469
Efficiency	5.31602	5.03906	3.73844	4.82109	4.83984	4.78125	4.88203	4.83281

PffBT4T2OD-PCBM

Parameter	Pixel 1	Pixel 2	Pixel 3	Pixel 4	Pixel 5	Pixel 6	Pixel 7	Pixel 8
Voc (V)	0.774514	0.769172	0.760899	0.758663	0.759806	0.755812	0.748985	0.724974
Jsc (mA/cr	-10.1172	-10.4922	-8.70703	-8.44531	-9.55078	-9.00781	-8.57422	-5.65234
FF (%)	46.9593	60.2895	47.3455	55.2691	57.9774	58.4026	56.0589	46.5456
Vmpp (V)	0.5	0.62	0.5	0.58	0.58	0.58	0.58	0.52
Jmpp (mA	-7.35938	-7.84766	-6.27344	-6.10547	-7.25391	-6.85547	-6.20703	-3.66797
Efficiency	3.67969	4.86555	3.13672	3.54117	4.20727	3.97617	3.60008	1.90734

11. References

1 X. Yi, C. H. Y. Ho, B. Gautam, L. Lei, A. H. Chowdhury, B. Bahrami, Q. Qiao and F. So, *J. Mater. Chem. C*, , DOI:10.1039/d0tc03969a.

2 P. Li, Y. Zhang, T. Yu, Q. Zhang, J.-P. Masse, Y. Yang, R. Izquierdo, B. Sun and D. Ma, *Sol. RRL*, 2020, **4**, 2000239.

3 H. Gaspar, F. Figueira, K. Strutyński, M. Melle-Franco, D. Ivanou, J. P. C. Tomé, C. M. Pereira, L. Pereira, A. Mendes, J. C. Viana and G. Bernardo, *Materials (Basel).*, 2020, **13**, 1267.

4 L. Duan, Y. Zhang, R. Deng, H. Yi and A. Uddin, *ACS Appl. Energy Mater.*, 2020, **3**, 5792–5803.

5 J. Zhang, W. Liu, M. Zhang, S. Xu, F. Liu and X. Zhu, *J. Mater. Chem. A*, 2020, **8**, 8661–8668.

6 M. Ren, G. Zhang, Z. Chen, J. Xiao, X. Jiao, Y. Zou, H. L. Yip and Y. Cao, *ACS Appl. Mater. Interfaces*, 2020, **12**, 13077–13086.

7 D. Yang, B. Cao, V. Körstgens, N. Saxena, N. Li, C. Bilko, S. Grott, W. Chen, X. Jiang, J. E. Heger, S. Bernstorff and P. Müller-Buschbaum, *ACS Appl. Energy Mater.*, 2020, **3**, 2604–2613.

8 X. Zhang, P. Fan, S. Hou, Y. X. Zheng and J. Yu, *Sol. Energy*, 2020, **198**, 535–541.

9 X. Yi, Z. Peng, B. Xu, D. Seyitliyev, C. H. Y. Ho, E. O. Danilov, T. Kim, J. R. Reynolds, A. Amassian, K. Gundogdu, H. Ade and F. So, *Adv. Energy Mater.*, 2020, **10**, 1902430.

10 L. Duan, Y. Zhang, H. Yi, F. Haque, C. Xu, S. Wang and A. Uddin, *Mater. Sci. Semicond. Process.*, 2020, **105**, 104750.

11 R. Zhao, B. Lin, J. Feng, C. Dou, Z. Ding, W. Ma, J. Liu and L. Wang, *Macromolecules*, 2019, **52**, 7081–7088.

12 L. Duan, H. Yi, Z. Wang, Y. Zhang, F. Haque, B. Sang, R. Deng and A. Uddin, *Sustain. Energy Fuels*, 2019, **3**, 2456–2463.

13 L. Arunagiri, G. Zhang, H. Hu, H. Yao, K. Zhang, Y. Li, P. C. Y. Chow, H. Ade and H. Yan, *Adv. Funct. Mater.*, 2019, **29**, 1902478.

14 T. Liu, W. Gao, G. Zhang, L. Zhang, J. Xin, W. Ma, C. Yang, H. Yan, C. Zhan and J. Yao, *Sol. RRL*, 2019, **3**, 1800376.

15 W. Zhang, R. Hu, X. Zeng, X. Su, Z. Chen, X. Zou, J. Peng, C. Zhang and A. Yartsev, *Polymers (Basel).*, 2019, **11**, 408.

16 Y. Zhang, M. T. Sajjad, O. Blaszczyk, A. J. Parnell, A. Ruseckas, L. A. Serrano, G. Cooke and I. D. W. Samuel, *Chem. Mater.*, 2019, **31**, 6548–6557.

17 M. Nazari, E. Cieplechowicz, T. A. Welsh and G. C. Welch, *New J. Chem.*, 2019, **43**, 5187–5195.

18 X. Zhang, P. Fan, Y. Han and J. Yu, *Energy Technol.*, 2019, **7**, 263–268.

19 L. Yan, Y. Wang, J. Wei, G. Ji, H. Gu, Z. Li, J. Zhang, Q. Luo, Z. Wang, X. Liu, B. Xu, Z. Wei and C. Q. Ma, *J. Mater. Chem. A*, 2019, **7**, 7099–7108.

20 B. Xu, I. Pelse, S. Agarkar, S. Ito, J. Zhang, X. Yi, Y. Chujo, S. Marder, F. So and J. R. Reynolds, *ACS Appl. Mater. Interfaces*, 2018, **10**, 44583–44588.

21 H. Tan, Y. Long, J. Zhang, J. Zhu, J. Yang, J. Yu and W. Zhu, *Dye. Pigment.*, 2019, **162**, 797–801.

22 J. Farinhas, D. Molina, A. Olcina, C. Costa, L. Alcácer, F. Fernández-Lázaro, Á. Sastre-Santos and A. Charas, *Dye. Pigment.*, 2019, **161**, 188–196.

23 Z. Liu and N. Wang, *J. Power Sources*, 2018, **402**, 333–339.

24 R. Singh, S. R. Suranagi, J. Lee, H. Lee, M. Kim and K. Cho, *Sci. Rep.*, 2018, **8**, 1–9.

25 Z. Bi, H. B. Naveed, Y. Mao, H. Yan and W. Ma, *Macromolecules*, 2018, **51**, 6682–6691.

26 C. Xu, M. Wright, D. Ping, H. Yi, X. Zhang, M. D. A. Mahmud, K. Sun, M. B. Upama, F. Haque and A. Uddin, *Org. Electron.*, 2018, **62**, 261–268.

27 C. Xu, M. Wright, N. K. Elumalai, M. A. Mahmud, V. R. Gonçales, M. B. Upama and A. Uddin, *J. Mater. Sci. Mater. Electron.*, 2018, **29**, 16437–16445.

28 G. Zhang, R. Xia, Z. Chen, J. Xiao, X. Zhao, S. Liu, H.-L. Yip and Y. Cao, *Adv. Energy Mater.*, 2018, **8**, 1801609.

29 M. Kim, J. Lee, D. H. Sin, H. Lee, H. Y. Woo and K. Cho, *ACS Appl. Mater.*

Interfaces, 2018, **10**, 25570–25579.

30 Y. Yan, W. Li, F. Cai, J. Cai, Z. Huang, R. S. Gurney, D. Liu, D. G. Lidzey, A. J. Pearson and T. Wang, *ACS Appl. Energy Mater.*, 2018, **1**, 3505–3512.

31 Y. Zhang, A. J. Parnell, O. Blaszczyk, A. J. Musser, I. D. W. Samuel, D. G. Lidzey and G. Bernardo, *Phys. Chem. Chem. Phys.*, 2018, **20**, 19023–19029.

32 J. Zhao, S. Zhao, Z. Xu, D. Song, B. Qiao, D. Huang, Y. Zhu, Y. Li, Z. Li and Z. Qin, *ACS Appl. Mater. Interfaces*, 2018, **10**, 24075–24081.

33 J. Wei, G. Ji, C. Zhang, L. Yan, Q. Luo, C. Wang, Q. Chen, J. Yang, L. Chen and C. Q. Ma, *ACS Nano*, 2018, **12**, 5518–5529.

34 C. Xu, M. Wright, N. K. Elumalai, M. A. Mahmud, D. Wang, V. R. Gonçales, M. B. Upama, F. Haque, J. J. Gooding and A. Uddin, *Appl. Phys. A Mater. Sci. Process.*, 2018, **124**, 449.

35 J. Zhao, D. Song, B. Qiao, Z. Xu, D. Huang, M. Wang, X. Zhang, Y. Li, Y. Zhu and S. Zhao, *Org. Electron.*, 2018, **58**, 178–184.

36 M. Xiao, K. Zhang, Y. Jin, Q. Yin, W. Zhong, F. Huang and Y. Cao, *Nano Energy*, 2018, **48**, 53–62.

37 T. Umeyama, K. Igarashi, D. Sakamaki, S. Seki and H. Imahori, *Chem. Commun.*, 2018, **54**, 405–408.

38 W. Li, J. Cai, F. Cai, Y. Yan, H. Yi, R. S. Gurney, D. Liu, A. Iraqi and T. Wang, *Nano Energy*, 2018, **44**, 155–163.

39 W. Li, Y. Yan, Y. Gong, J. Cai, F. Cai, R. S. Gurney, D. Liu, A. J. Pearson, D. G. Lidzey and T. Wang, *Adv. Funct. Mater.*, 2018, **28**, 1704212.

40 G. Ji, Y. Wang, Q. Luo, K. Han, M. Xie, L. Zhang, N. Wu, J. Lin, S. Xiao, Y. Q. Li, L. Q. Luo and C. Q. Ma, *ACS Appl. Mater. Interfaces*, 2018, **10**, 943–954.

41 H. Cha, S. Wheeler, S. Holliday, S. D. Dimitrov, A. Wadsworth, H. H. Lee, D. Baran, I. McCulloch and J. R. Durrant, *Adv. Funct. Mater.*, , DOI:10.1002/adfm.201704389.

42 G. Pirotte, S. Agarkar, B. Xu, J. Zhang, L. Lutsen, D. Vanderzande, H. Yan, P. Pollet, J. R. Reynolds, W. Maes and S. R. Marder, *J. Mater. Chem. A*, 2017, **5**, 18166–18175.

43 H. Cha, J. Wu, A. Wadsworth, J. Nagitta, S. Limbu, S. Pont, Z. Li, J. Searle, M. F. Wyatt, D. Baran, J. S. Kim, I. McCulloch and J. R. Durrant, *Adv. Mater.*, , DOI:10.1002/adma.201701156.

44 J. Czolk, D. Landerer, M. Koppitz, D. Nass and A. Colsmann, *Adv. Mater. Technol.*, 2016, **1**, 1600184.

45 Q. Sun, F. Zhang, Q. An, M. Zhang, X. Ma and J. Zhang, *ACS Appl. Mater. Interfaces*, 2017, **9**, 8863–8871.

46 F. Zhao, Y. Li, Z. Wang, Y. Yang, Z. Wang, G. He, J. Zhang, L. Jiang, T. Wang, Z. Wei, W. Ma, B. Li, A. Xia, Y. Li and C. Wang, *Adv. Energy Mater.*, 2017, **7**, 1602552.

47 Q. Sun, F. Zhang, Q. An, M. Zhang, J. Wang and J. Zhang, *Phys. Chem. Chem. Phys.*, 2017, **19**, 709–716.

48 Z. Jiang, *J. Appl. Crystallogr.*, 2015, **48**, 917–926.

49 J. C. Blakesley, F. A. Castro, W. Kylberg, G. F. A. Dibb, C. Arantes, R. Valaski, M. Cremona, J. Soo and J. Kim, *Org. Electron.*, 2014, **15**, 1263–1272.

50 K. Sasitharan, D. G. Bossanyi, N. Vaenas, A. J. Parnell, J. Clark, A. Iraqi, D. G. Lidzey and J. A. Foster, *J. Mater. Chem. A*, , DOI:10.1039/C9TA12313J.

51 M. Zhao, Y. Wang, Q. Ma, Y. Huang, X. Zhang, J. Ping, Z. Zhang, Q. Lu, Y. Yu, H. Xu, Y. Zhao and H. Zhang, *Adv. Mater.*, 2015, **27**, 7372–7378.
